# Supplementary material for: A comparison of 27 Arabidopsis thaliana genomes and the path toward an unbiased characterization of genetic polymorphism
Source: Nat Genet. 2025 Aug 19;57(9):2289–301. doi: 10.1038/s41588-025-02293-0 (PMC12425826; doi:10.1038/s41588-025-02293-0)
Supplement: Supplementary file 1 — Supplementary Notes 1–8 and Figs. 1–33. [file 41588_2025_2293_MOESM1_ESM.pdf]

nature genetics

Supplementary information https://doi.org/10.1038/s41588-025-02293-0

A comparison of 27 *Arabidopsis thaliana* genomes and the path toward an unbiased characterization of genetic polymorphism

In the format provided by the authors and unedited

1 Table of contents

1 Table of contents **1**

2 List of supplementary figures **2**

3 Genome assembly **3**
3.1 The organization of genome variation 3
3.2 Reciprocal translocation in 22001 3
3.3 Genome size estimation 5
3.4 Estimation of satellite repeats 5
3.5 Organellar insertions 6

4 Comparing Pannagram and PGGB **8**

5 The mobile-ome **12**
5.1 sSVs and annotated TEs 12
5.2 Un- or mis-annotated TE families 12
5.3 Silencing of sSVs 18

6 The gene-ome **21**
6.1 Details about reconciling annotations and gene filtering 21
6.2 Genes and TEs 22
6.3 New genes 23

7 Errors and biases in SNP-calling **27**
7.1 Results 27
7.2 Methods and parameters 27

8 Errors and biases in DNA methylation profiling **33**

2 List of supplementary figures

1 The causes of contig breaks . . . 3
2 Scaffolded vs. unplaced contigs . . . 4
3 TE class size distribution . . . 4
4 Reciprocal translocation in accession 22001 . . . 5
5 PCR-validation of reciprocal translocation in 22001 . . . 6
6 Geneome size vs. tandem repeats . . . 6
7 Organellar inserts . . . 7
8 Comparing SVs from Pannagram and PGGB . . . 8
9 Why graph SVs are longer than Pannagram SVs . . . 9
10 SVs and closely linked duplications . . . 10
11 SVs in regions that are difficult to align . . . 11
12 TE-superfamily content in sSVs . . . 12
13 Different TE annotations of very similar mobile elements . . . 13
14 A mobile element containing annotated TEs . . . 14
15 Another element containing annotated TEs . . . 15
16 Un-annotated TE suggesting horizontal transfer . . . 16
17 A putative novel mobile element family . . . 17
18 Methylation of sSVs . . . 19
19 Expression of sSV sequences . . . 20
20 Reconciling ambiguous gene annotation with majority voting . . . 21
21 Variation in gene models . . . 22
22 Number of segregating genes . . . 22
23 An example of the final consensus annotation . . . 23
24 TEs in *de novo* annotation. . . . 23
25 ‘New’ genes . . . 24
26 Silencing of ‘new” genes . . . 26
27 Sources of errors in SNP-calling . . . 28
28 Failure of alignment causes both FP and FN calls . . . 29
29 How duplications introduce FP calls . . . 30
30 How duplications introduce FN calls . . . 31
31 Duplications contribute to the majority of mis-called heterozygous SNPs . . . 32
32 Reference bias: methylation. . . . 34
33 Original (top) and inverted gel images corresponding to Supplementary Fig. 5 . . . 35

3 Genome assembly

3.1 The organization of genome variation

We sequenced the 27 genomes sequenced with PacBio continuous long reads (CLRs) to high depth, assembled them into contigs with Canu[77], and polished these contigs with PCR-free short reads[114] (for assembly statistics, see Supplementary Table 1). To reconstruct chromosomes, we generated hybrid scaffolds with individual Bionano optical maps for eight accessions, which allowed us to determine the most appropriate parameters for scaffolding[79] based on the TAIR10 reference genome[115]. Although this potentially introduces some reference bias, our focus is on the gene-dense chromosome arms, which are well-covered by large contigs, and appear to harbor few large-scale rearrangements.

Like many plants, *A. thaliana* has experienced recent episodes of TE activity, leading to nearly identical sequences inserted across each genome[38]. These make short-read alignments difficult, but the PacBio CLR technology used here produced reads long enough to bridge such insertions. However, extensive tracts of identical or near-identical tandem repeats, such as centromere satellites and 5S rDNAs, consistently break our assemblies (Supplementary Figs. 1-2). We note that centromeres can now be assembled with PacBio HiFi reads[16]. CLRs are less accurate, but they are on average longer, hence marginally better for chromosome arms[75]. 45S rDNA clusters remain challenging regardless of technology[39].

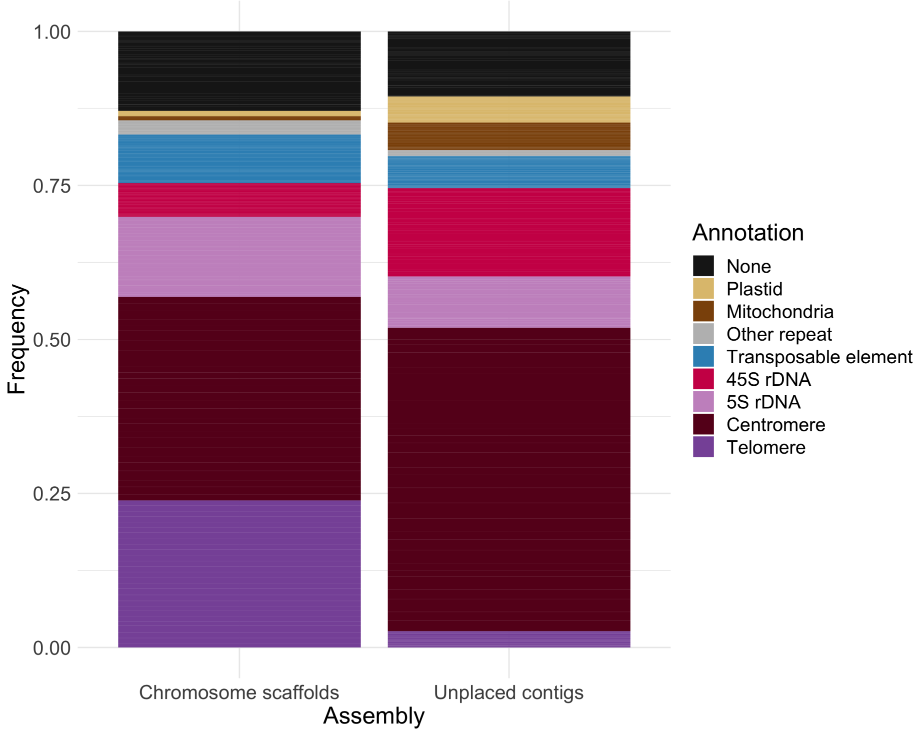

Supplementary Fig. 1 | The causes of contig breaks. Stacked bar chart summarizing the type of repetitive element closest to each contig edge across the 27 assemblies, separately for scaffolded and unplaced contigs. Seventy-two percent of the latter end with centromeric or rDNA repeats.

Our 27 assemblies are all ∼120 Mb in size, whereas the full genomes, consistent with previous results[40,41], are estimated to range from 135 to 155 Mb (Fig. 1b). A BLAST-based approach indicates that centromeres and rDNA clusters alone account for up to 92% of the estimated variation (Fig. 1b-c), with the importance of 45S rDNA variation having been appreciated before[41]. While individual TE families can vary greatly in size across accessions (Supplementary Fig. 3), we confirm that the cumulative effect of all TEs on genome size variation appears to be small in *A. thaliana*[41]—contrary to the major role they play in inter-specific variation[42,43].

3.2 Reciprocal translocation in 22001

We discovered a very large reciprocal translocation in accession 22001 (alternative name 85-3) from the Yangtze River region, which swapped the distal portions of chromosomes 3 and 5 (Supplementary Fig. 4). We validated the translocation by PCR

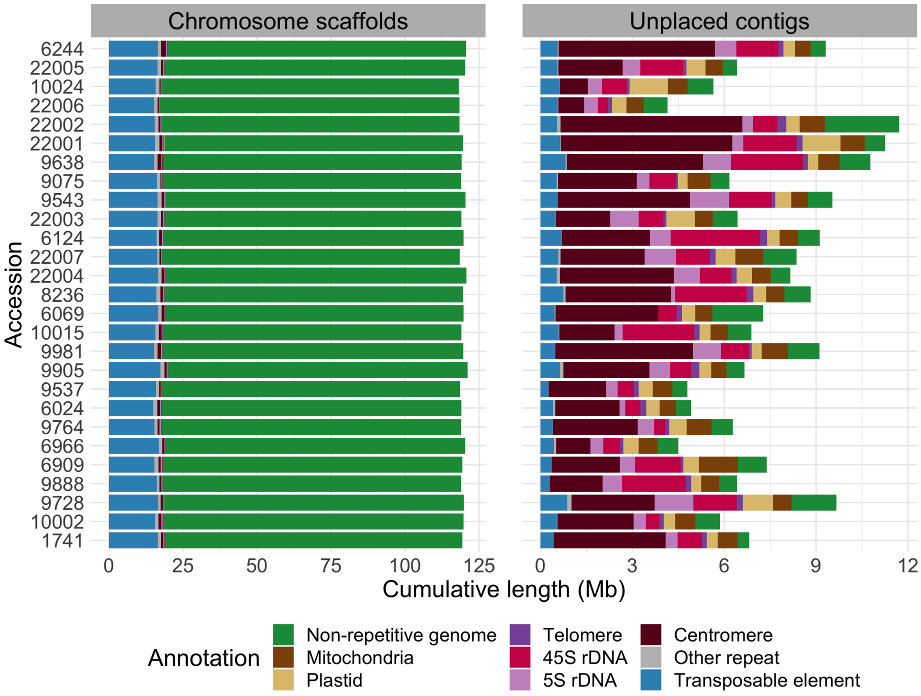

Supplementary Fig. 2 | Scaffolded vs. unplaced contigs. The former correspond to the chromosome arms and contain mostly non-repetitive sequence and TEs, while the latter mostly contain centromeric and rDNA repeats, as well as organellar DNA sequence (*cf.* Fig. 1).

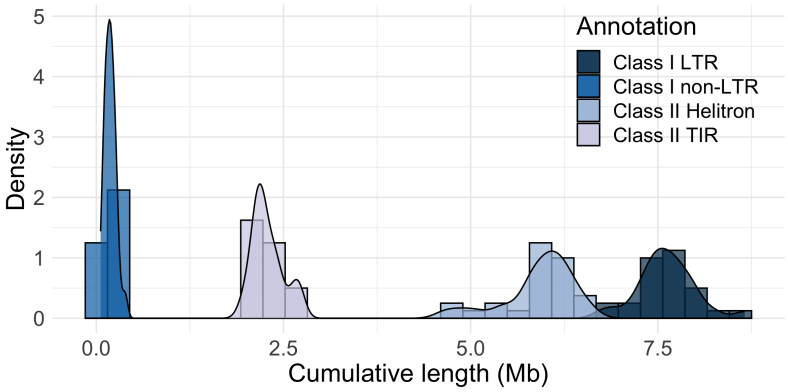

Supplementary Fig. 3 | TE class size distribution. The cumulative length of all copies of a given TE class differs greatly across accessions, but the total TE content does not.

with two sets of primer pairs designed to either amplify the standard arrangement of chromosomes 3 and 5 of Col-0, or the two translocation junction regions in accession 22001 (Supplementary Fig. 5). This rearrangement, which would presumably lead to decreased fertility in heterozygotes, appears to be quite rare as we did not identify other examples in a sample of 117 accessions sequenced with short reads from the same region[35]. For the purposes of this study, we manually rearranged this genome to match the ancestral organization. To identify the exact breakpoints, we aligned chromosome 3 of 22001 to all other sequences of chromosome 3 with minimap2 (`-x asm5`). After filtering the alignments to retain those longer than 50 kb (`fpa drop -l 50000`), we removed the sequence from the start to the first position of alignment and added the reverse complement to the end of chromosome 5. A collection of the scripts can be found at the project GitHub repository.

Similar steps were followed for the segment in chromosome 5, using the sequence starting at the last position of the alignment to the end of the originally assembled chromosome. The sequence was removed from chromosome 5 and added to the beginning of chromosome 3.

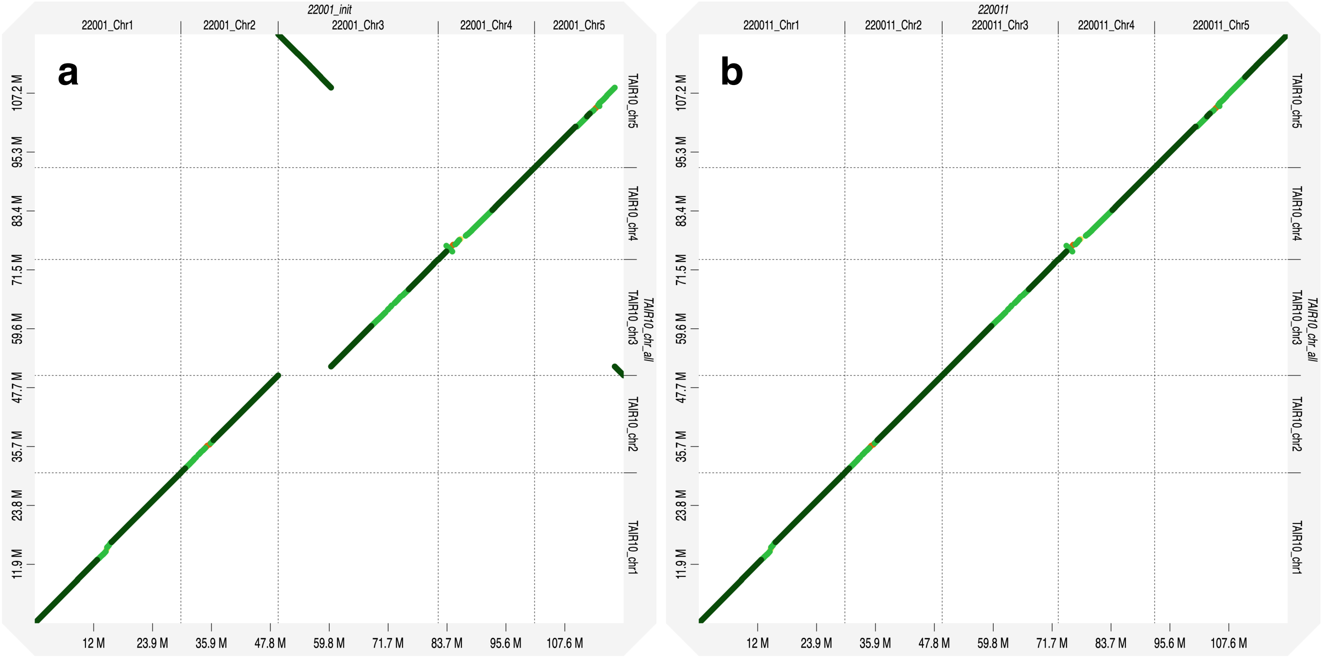

Supplementary Fig. 4 | Reciprocal translocation in accession 22001. Dot plot of the original assembly 22001 (**a**) and of the modified assembly 22001m (**b**) against the TAIR10 genome. The translocation is readily seen at the beginning of chromosome 3 and the end of 5. Dot plots were created with D-GENIES.

We searched the repeat annotation for clues as to what type of sequence might have been responsible for the translocation, but found no obvious cause.

3.3 Genome size estimation

To estimate genome sizes from PCR-free reads, we employed a k-mer based approach[90] after pre-processing the datasets. First, we trimmed adapters from the raw reads and removed low-quality sequences with cutadapt v2.4[108] (`-q 20,15 -trim-n -minimum-length 75`). We aligned the trimmed reads to the organellar genomes of TAIR10 and the bacteriophage phiX174 genome with bwa-mem v0.7.17[116], and executed a series of samtools v1.9[117] commands to keep only reads for which both pairs did not align to any of these genomes. Briefly, we used `samtools view -b -f 12 -F 256` to obtain unmapped read pairs; `samtools view -b -f 4 -F 264` for paired-reads alignments in which read1 was unmapped and read2 was mapped; and `samtools view -b -f 8 -F 260` paired-read alignments in which read1 was mapped and read2 was unmapped. Then, we combined the three outputs of the described steps with `samtools merge`, discarded supplementary alignments with `samtools view -b -F 2048` and converted the BAM file to FASTQ format with `bedtools bamtofastq`. To avoid biases due to different read lengths for all subsequent analyses, we trimmed reads in all data sets to the common minimum of 124 bp and removed reads shorter than that with cutadapt v2.4[108] (`-length 124 -minimum-length 124`). Finally, we counted 21 bp long k-mers with the commands `count -C -m 21 -s 5G` and `histo` from Jellyfish v2.3.0[118], and the outputs were processed by the findGSE tool[119] to estimate genome sizes (Fig. 1).

3.4 Estimation of satellite repeats

To estimate the contribution that the three main classes of satellite repeats make to the genomes — centromere, 45S and 5S rDNA repeats — from PCR-free short reads, we used BLAST v2.2.29[120] (`blastn -evalue 1e-10 -soft_masking false -dust no -max_hsps 1 -outfmt`), with reads trimmed to 124 bp in length as queries (see above) against a database with representative sequences of some of the most abundant or genetically diverse CEN159 and CEN178 satellite repeats collected from a recent pancentromere study[16], as well as three consensus 5S rDNA units[82], and a reference 45S rDNA copy[83]. For each repeat class, the full-length of reads with a blast hit was added, and their sum was divided by the coverage estimated by `findGSE`. The sum of centromere and rDNA repeats estimated by this method and the difference

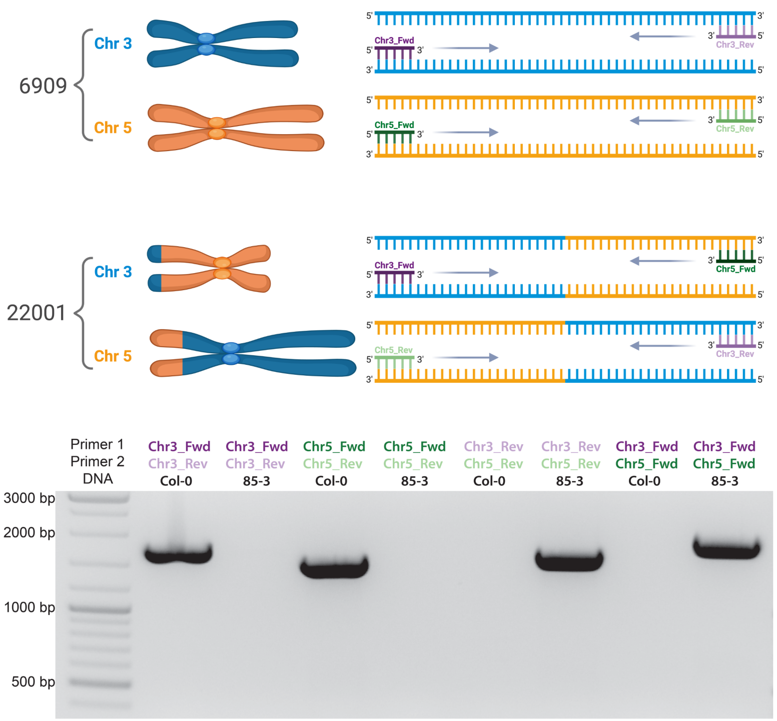

Supplementary Fig. 5 | Validation by PCR of the reciprocal translocation in accession 22001.

between genome size (estimated by the k-mer approach) and the scaffolded assembly were highly correlated ($r^2 = 0.9639$; $P < 2.2 \times 10^{-16}$; see Supplementary Fig. 6).

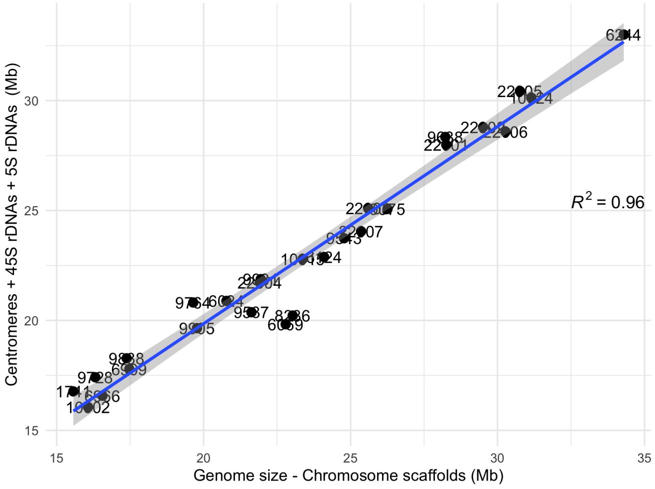

Supplementary Fig. 6 | Correlation between the combined sum of centromere and rDNA repeats estimated by a BLAST-based approach with PCR-free reads, and the difference between the total genome size estimated by a k-mer approach with PCR-free reads and the summed lengths of the chromosome scaffolds.

3.5 Organellar insertions

We identified insertions of organellar DNA in the nuclear genomes by aligning the organellar genomes from the TAIR10 reference[115] with minimap2 v2.16[121] (`-cx asm5`). We found 108 insertions, almost all of them singletons or doubletons,

ranging from a few hundred bp to entire organellar genomes (Supplementary Fig. 7, Supplementary Table 3). None of our genomes, other than our strain of the reference accession Col-0, 6909, shared the large nuclear insertion of mitochondrial DNA in chromosome 2 of the TAIR10 reference[75,122,123].

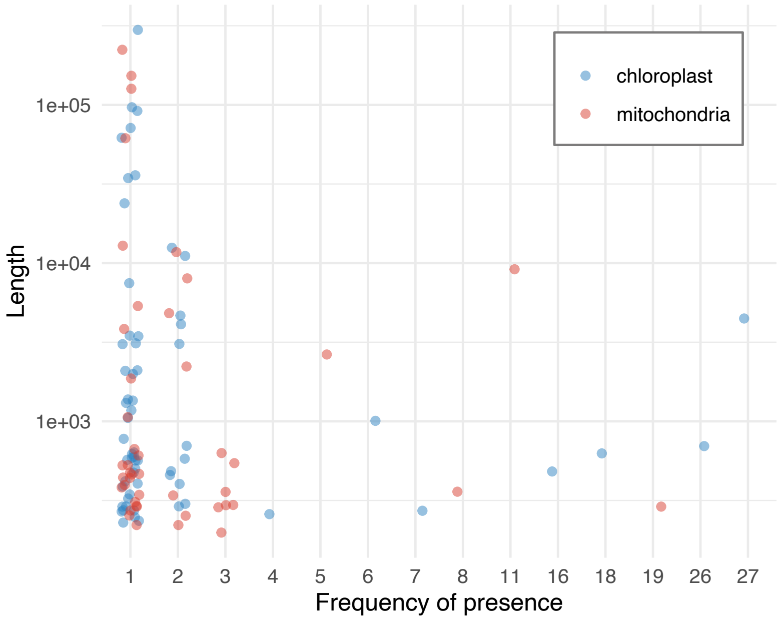

Supplementary Fig. 7 | Frequency and size distribution of organellar insertions. Seven insertions are excluded because they were associated with contig breaks, and we could therefore not determine their exact length (Supplementary Fig. 1).

4 Comparing Pannagram and PGGB

Comparing the SVs identified by two conceptually different approaches was not straightforward. SVs identified by Pannagram were typically covered by PGGB variants, which included nearly twice as much sequence, especially in highly polymorphic pericentromeric regions (Supplementary Fig. 8). A trivial reason for this difference is that Pannagram masked centromeric regions full of tandem repeat arrays (Fig. 2a-c), but we also identified several less obvious causes (see Supplementary Figs. 9–11 for details and examples).

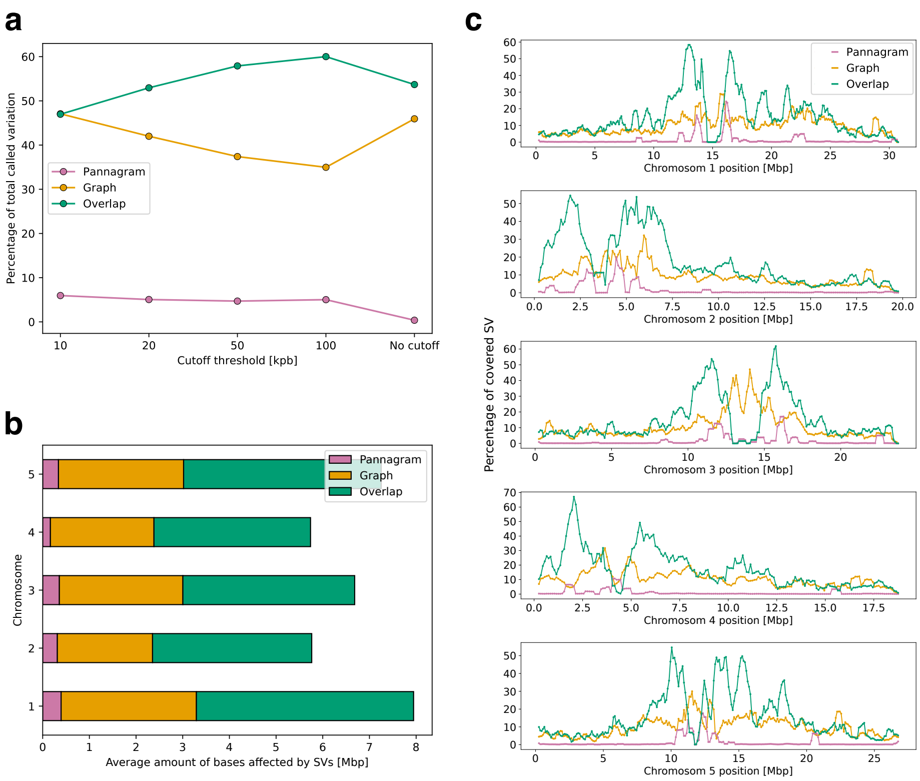

Supplementary Fig. 8 | Comparing SVs from Pannagram and PGGB graphs. a, Scatter plot of overlap and method-specific SVs as a function of eliminating SVs above a certain length-cutoff. Overall, the overlap between the two methods is 50%, but the overlap can be increased by removing large SVs (demonstrating that disagreement is disproportionally due to large SVs. **b,** Comparison of Pannagram and graph-based SVs across chromosomes (average per accession), demonstrating that there are no major differences between chromosomes. SVs shorter than 15 bp were not included in this figure. **c,** Position of overlapping and method-specific SVs for each chromosome of accession 6909 (Col-0). Large discrepancies are more pronounced close to the centromeres (there is no overlap inside centromeres, as these are masked by Pannagram). Each dot represents a 100 kb window, using a moving average of five windows.

First, the presence of physically distant but closely related sequences (*e.g.*, reflecting recent TE activity) can lead to large loops in the PGGB graph that do not reflect actual SVs. Masking repetitive sequences will reduce this problem, but requires good repeat annotation—and would also make it impossible to study genome-variation comprehensively (one of our goals in this paper). Second, even in the case of tandem duplications, the graph combines duplicated sequences into a single node and hence counts all these sequences as part of SVs, even if not all of them are variable. Third, PGGB and Pannagram rely on different alignment parameters. To reduce the number of uninterpretable nodes, PGGB requires strict similarity criteria, whereas Pannagram can use more relaxed cutoffs to maximize homology detection. The *A. thaliana* genome contains many highly diverged regions[59], and these tend to be treated by PGGB as long SVs, whereas Pannagram often finds short alignments, resulting in local clusters of shorter variants. Whether Pannagram or PGGB results are more biologically relevant ultimately depends on the question and the cause of high divergence.

A final difference between PGGB and Pannagram lies in how nested length variants are represented. PGGB shows these as easily interpreted loops-within-loops, whereas Pannagram treats them as complex SV regions. This does not affect the size of the region covered by SVs, but does cause differences in the SV counts.

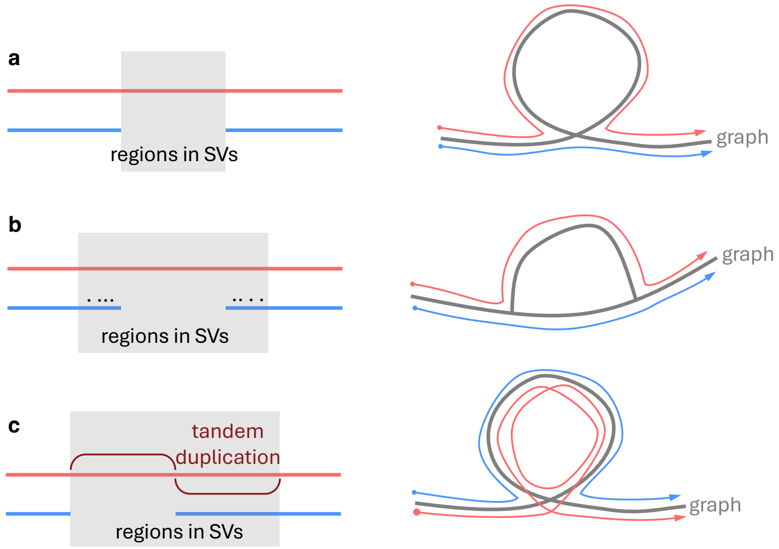

Supplementary Fig. 9 | Cartoons illustrating cases where graph SVs are longer than Pannagram SVs. a, Two genomes (red and blue) differ by a single simple SV (Extended Data Fig. 2a), which can be represented as a gap in the alignment, or a loop in the graph. Pannagram and the graph give the same result, and the length of the SV (indicated in grey) is identical. **b,** However, if SNPs, represented by dots, are linked to the SV, causing imperfect alignment in the flanking regions around the SV, PGGB may recognize longer haplotypes, resulting in an arrangement that resembles a hat. In this case, the graph SV is not merely a presence-absence variant, but a complex SV with two alleles. The entire region affected (in grey) is longer than the SV recognized by Pannagram (still the same as in A). **c,** When the SV is formed by a tandem duplication, the graph representation of the SV is topologically similar to scenario A, but the SV covers both the original sequence and its duplicated copy (grey region), while the SV identified by Pannagram is still the same as in A.

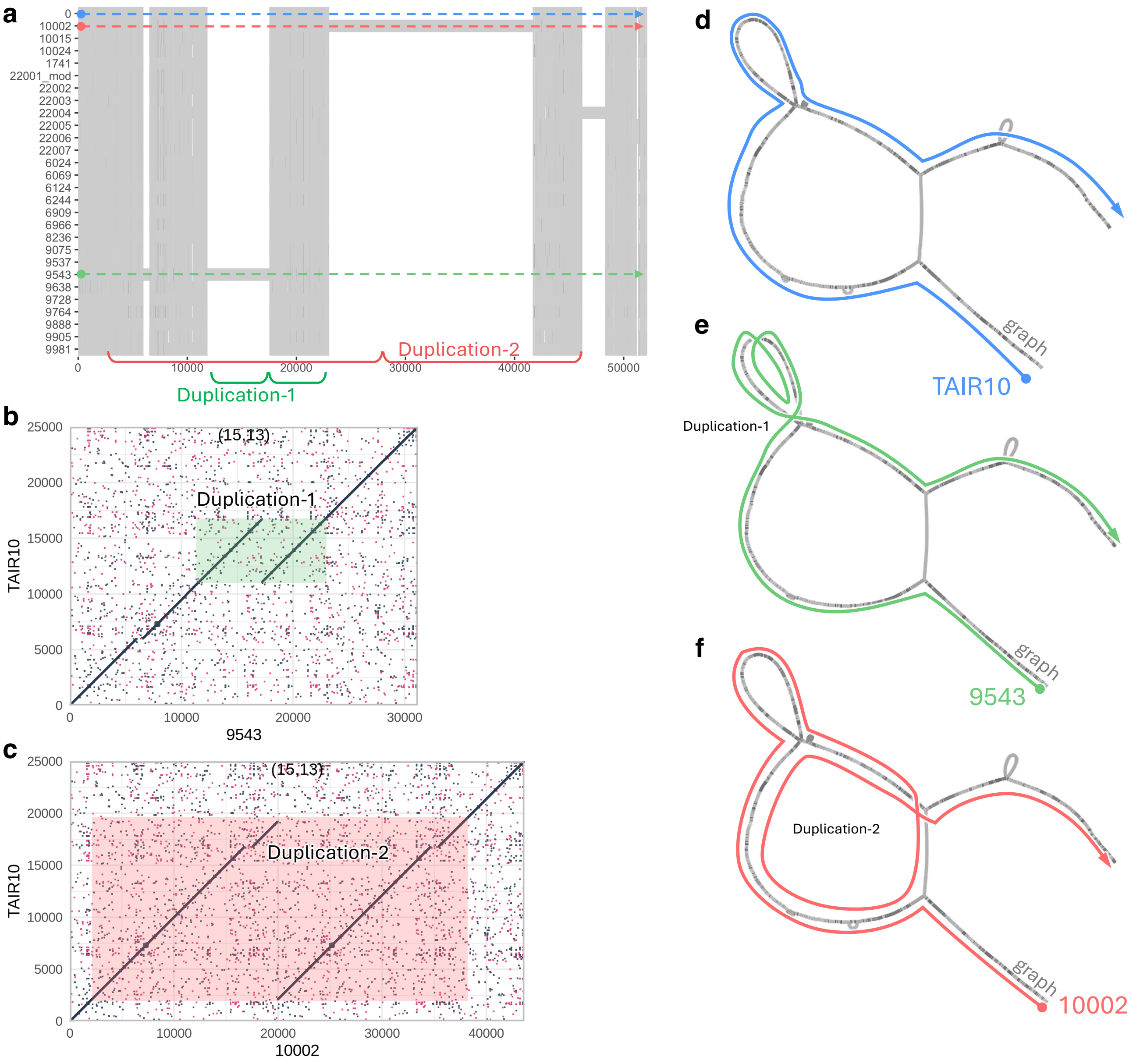

Supplementary Fig. 10 | An example of how Pannagram and the PGGB graph each handle closely linked duplications. The region depicted corresponds to coordinates 285,000-310,000 bp on chromosome 1 in accession 1741. The Pannagram alignment (panel **a**) identifies four simple SVs, with the two longest ones being due to duplicated sequences in accession 9543 (panel **b**) and accession 10002 (panel **c**). The PGGB graph representation of this region is shown on the right, along with paths corresponding to three different haplotypes. Panel **d** shows the path taken by accession 0 (TAIR10), which carries the majority haplotype. Panel **e** shows the path of accession 9543, which carries a duplication, and hence goes around the small loop twice. Panel **f** shows the path of accession 10002, which has the longest duplication, and hence goes around the big central loop twice. Thus, while Pannagram identifies four simple SVs (the longest one being 18.6 kb long), the PGGB graph SVs involve all accessions and cover almost the entire region shown. Note that, as in the cartoon example (Supplementary Fig. 9), similar PGGB graph topologies may result from very different types of sequence differences between accessions.

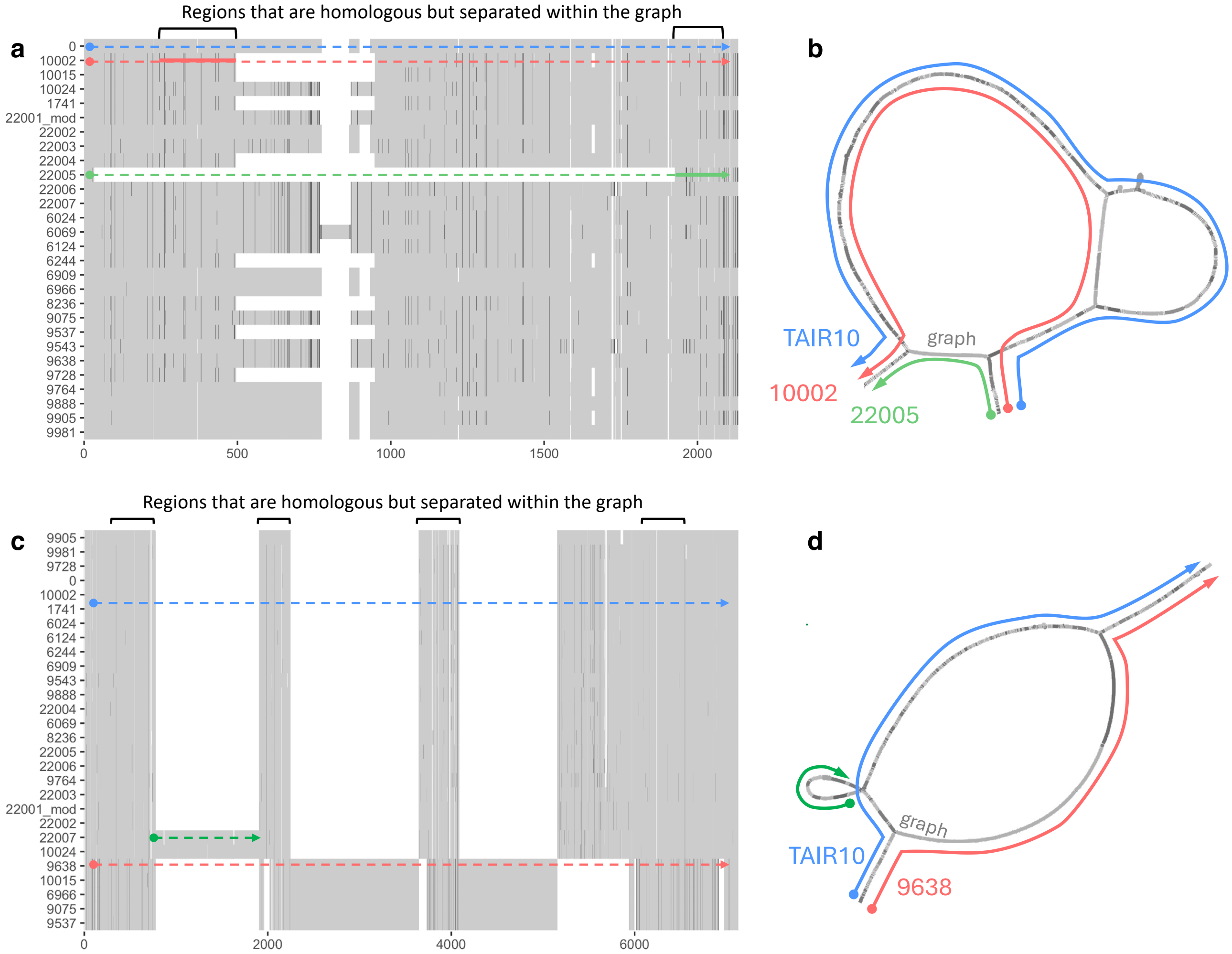

Supplementary Fig. 11 | Another example of how Pannagram and the PGGB graph each handle regions that are difficult to align. a, Pannagram alignment of a highly polymorphic region corresponding to coordinates 21,956,400-21,958,060 bp on chromosome 1 in accession 1741. Pannagram identifies a complex SV covering most of the region. **b,** The PGGB graph also recognizes these SVs, but merges them with flanking SNP variation, resulting in two nested hat-like structures (*cf.* Supplementary Fig. 9b). As a result, the sequence covered by SVs is longer. **c,** Pannagram alignment of the region corresponding to coordinates 1,183,130-1,186,590 bp on chromosome 1 in accession 0 (TAIR10). Pannagram identifies several, mostly simple SVs separated by short alignable regions. **d,** The PGGB graph does not align these regions, and merges most variants into two longer haplotypes. In this case as well, the graph SVs cover more sequence than the Pannagram SVs.

5 The mobile-ome

5.1 sSVs and annotated TEs

We showed that most sSVs correspond to different categories of overlap with annotated TE sequences (Fig. 4). This analysis can broken down further to the level of TE superfamilies. Different TE superfamilies show very different patterns with respect to these overlap categories, presumably reflecting both the biology of the superfamilies and the quality of the annotation (Supplementary Fig. 12a). That there is a difference between the categories can also be seen in a Principal Components analysis, where the two first PCs of TE superfamily composition distinguish four TE-content categories, consistent with different mechanisms underlying formation of different groups of TEs (Supplementary Fig. 12b).

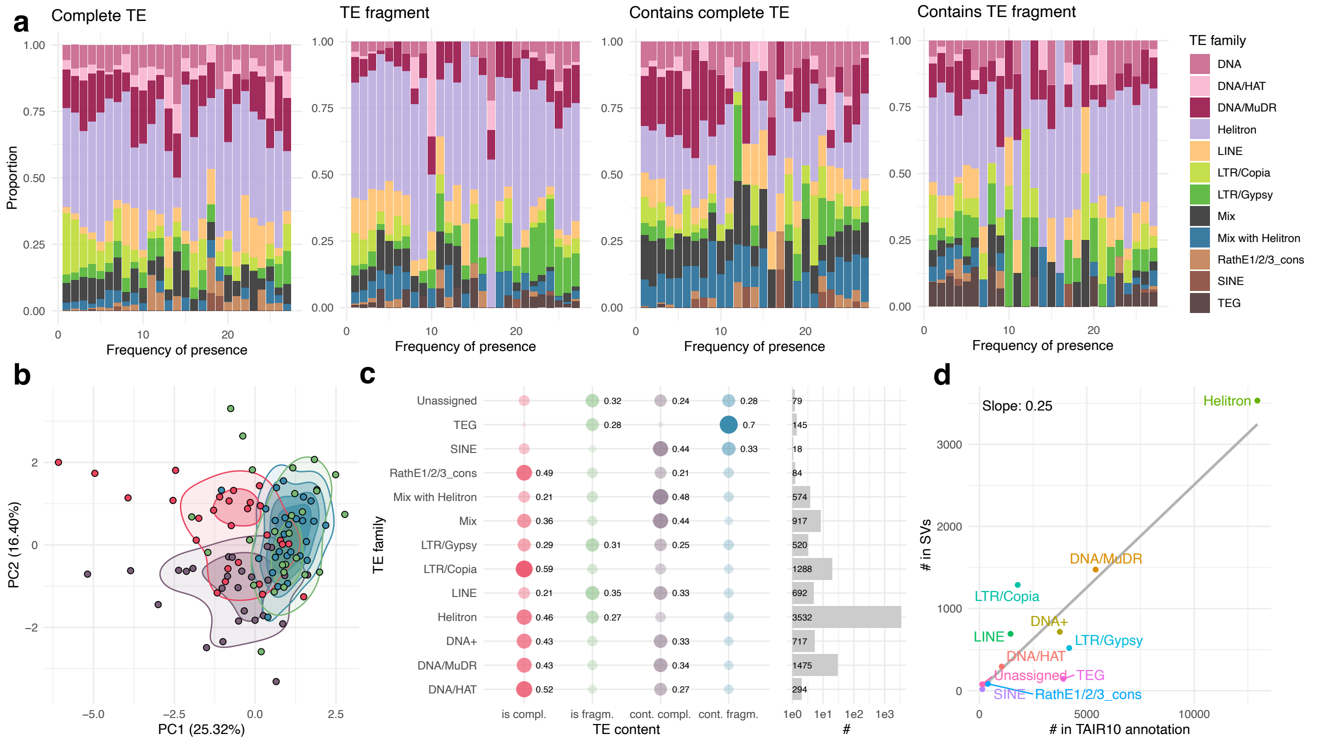

Supplementary Fig. 12 | TE-superfamily content in sSVs. a, Distribution of absolute numbers of sSVs across different TE content categories, based on the frequency of the presence allele. **b,** To confirm that the distributions in **(a)** differed, all columns in these subplots were taken as observations, and a PCA was performed. The second PC described the difference between categories of TE content based on their TE-superfamily content, in support of differences in TE-superfamily content in sSVs from different TE-overlap categories. **c,** Normalized distributions of TE-superfamily content according to the frequency of presence for each category of TE overlap. **d,** Correspondence between the number of TE superfamily members in the TAIR10 annotation and the representation of TE superfamilies in sSVs.

We found several differences between TE superfamilies. For example, LTR/Copia elements appear to be both active and fairly well annotated, with roughly 50% of matches corresponding to presence-absence polymorphisms that include apparently complete elements, whereas matches to LINE elements rarely correspond to complete elements (Supplementary Fig. 12c). Also notable is the relationship between presence in sSVs and representation in the annotation. In general, they are strongly correlated (Supplementary Fig. 12d), but LTR/Copia elements, for example, seem over-represented in sSVs, consistent with their continuing to be active[38].

5.2 Un- or mis-annotated TE families

Supplementary Figs. 13–17 show several examples of un- or mis-annotated TE families that are discussed in the main article.

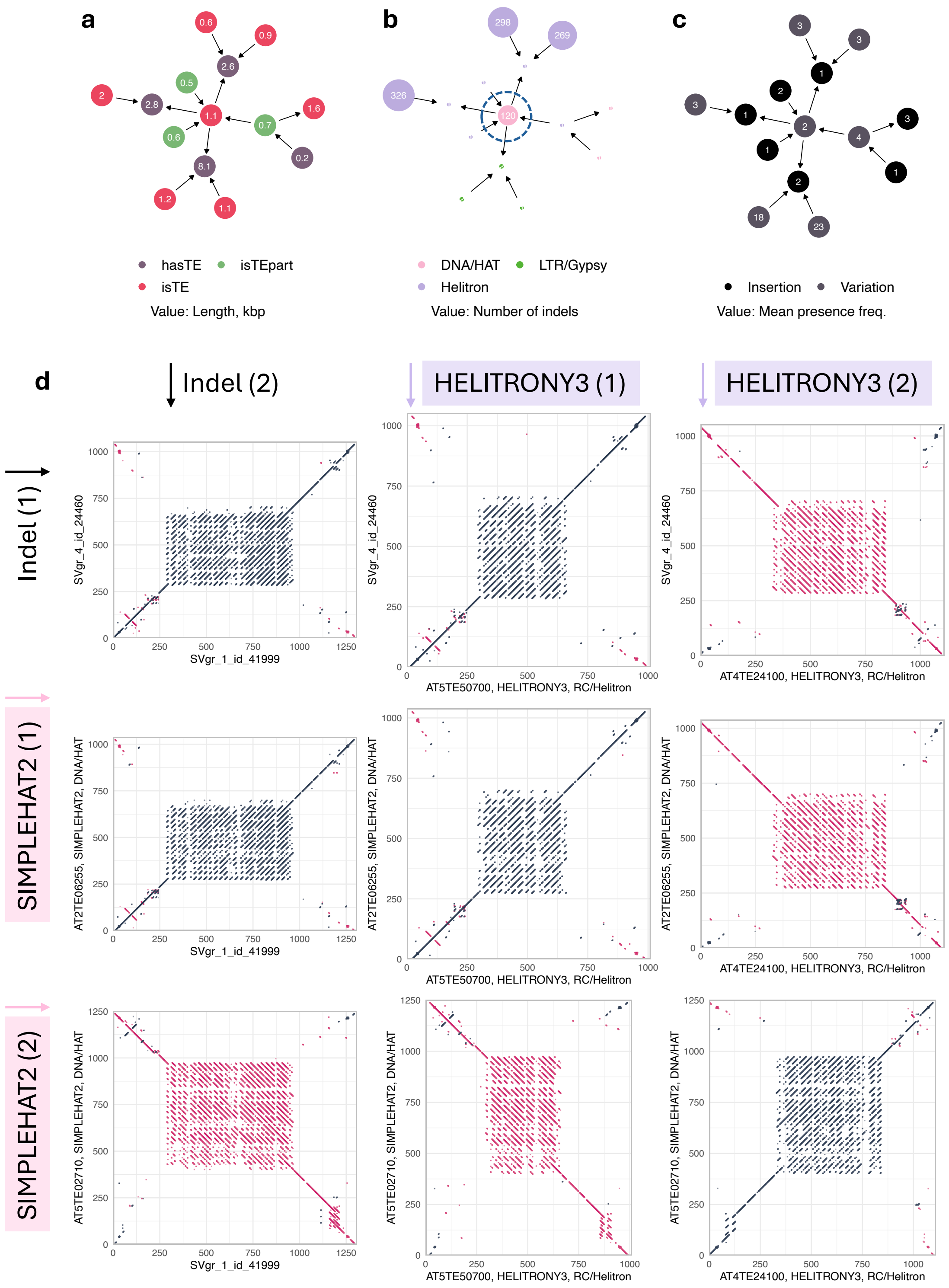

Supplementary Fig. 13 | Different TE annotations of very similar mobile elements. a–c, A specific part of our graph of nestedness (Fig. 5) colored separately by TE content, TE superfamily, and presence frequency. **d,** Dot plots comparing sSVs from the central node of the graph. Dark color reflects the similarity on the forward strand, pink color – on the reverse complement. The dot plots were constructed with a window parameter of 15 and the number of matches set to 12. Sequences in the central node are very similar to members of both HELITRONY3 and SIMPLEHAT2 families, which are also similar to each other, demonstrating how confusing TAIR10 TE annotation leads to nodes connecting different TE superfamilies.

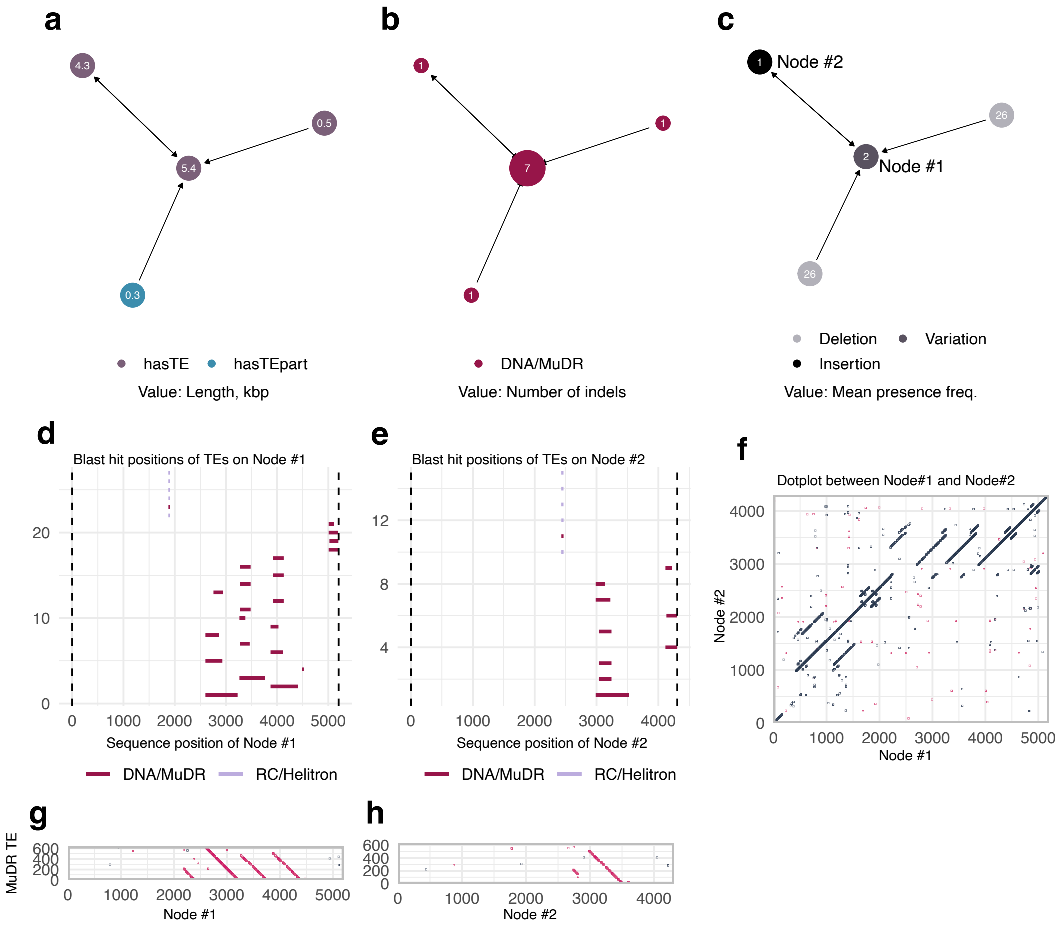

Supplementary Fig. 14 | An example of a mobile element containing annotated TE sequences. a–c, A connected component from the graph of nestedness (Fig. 5) colored separately by TE content, TE superfamily, and frequency (indicating likely insertion/deletion status). The component is characterized by putative insertions of a large element (5.4 kb) containing annotated DNA/MuDR element. As illustrated by BLAST results (**d–e**) and dot plots (**f–h**), the Node #1 presence alleles contains several copies of an annotated MuDR element, while the Node #2 allele contains one copy. The nature and mechanism of transposition of this mobile element is unclear.

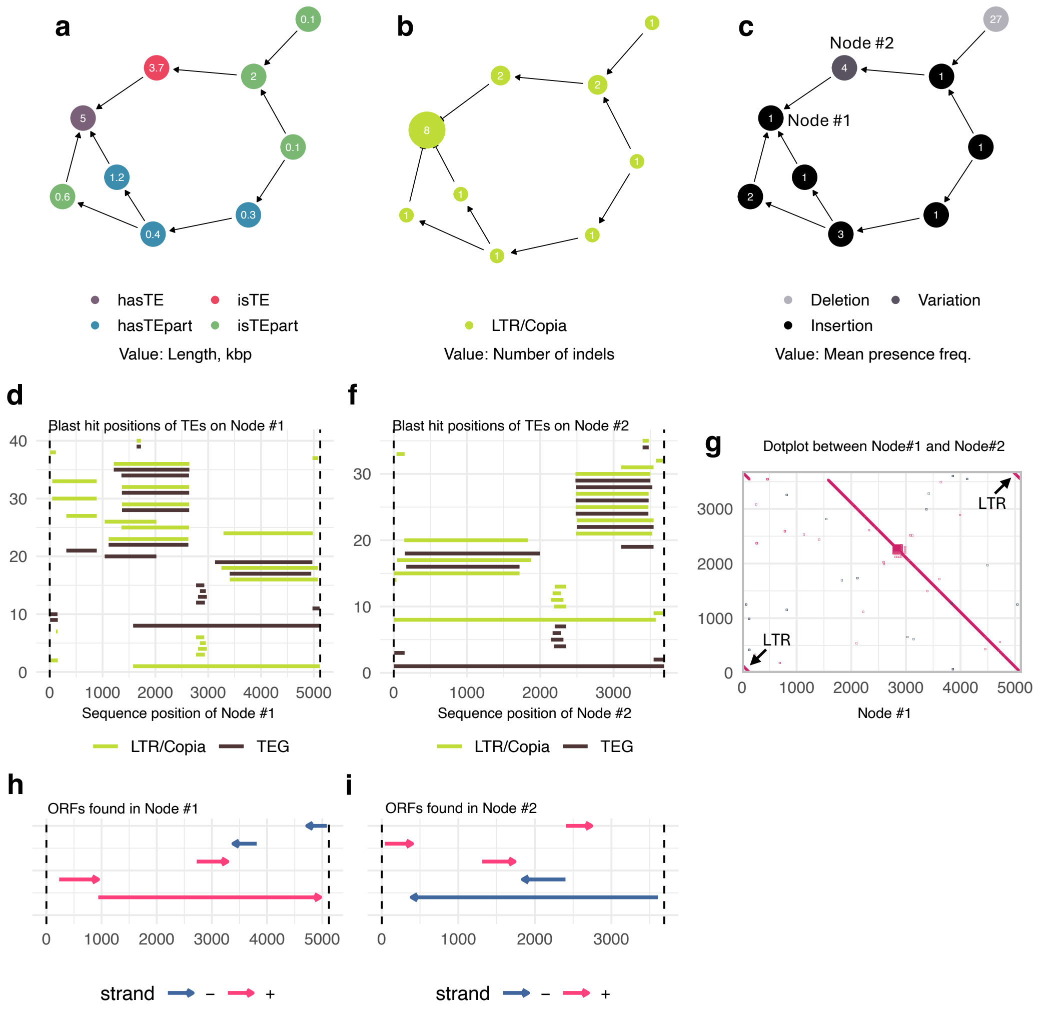

Supplementary Fig. 15 | Another example of a mobile element containing annotated TE sequences. a–c, A connected component from the graph of nestedness (Fig. 5) separately colored by TE content, TE superfamily, and frequency (indicating likely insertion/deletion status). **d–f,** The component is characterized two active elements, a 3.7 kb one (Node #2) corresponding to an annotated LTR/Copia element, and an apparently more active one (based on copy number) that is larger (5 kb, Node #1) and contains a very similar LTR/Copia element plus additional sequence, including LTR/Copia fragments. Both elements have (matching) LTRs (**g**), as well as LTR/Copia ORFs (**h–i**). Unlike the example in Supplementary Fig. 14, the nature and mechanism of this element are clearer, and we have previously described an LTR/Copia element that is longer than the existing TAIR10 annotation suggests[48].

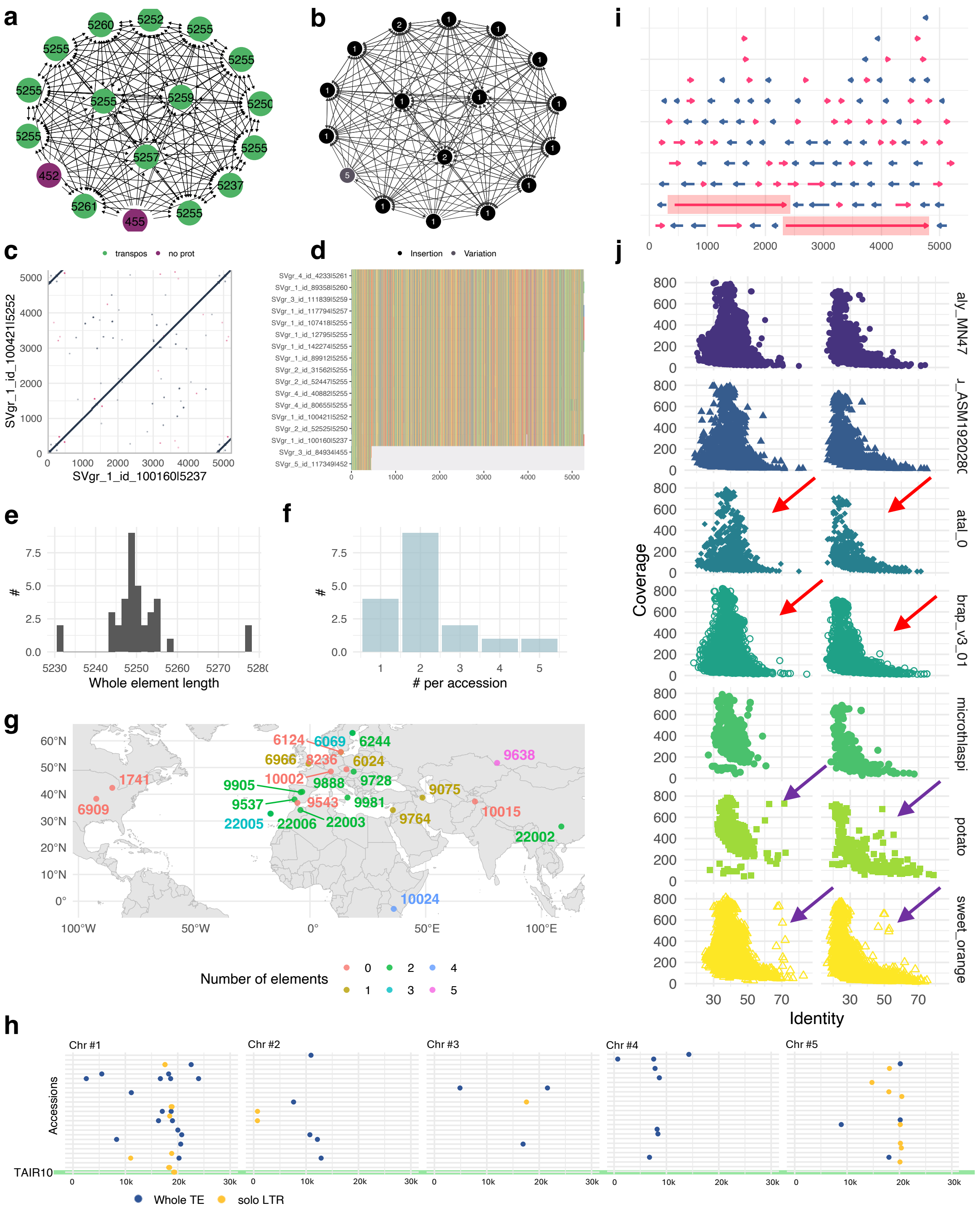

Supplementary Fig. 16 | An un-annotated TE with evidence of horizontal transfer. A graph component built on sSVs that have no overlap with annotated TEs consists of many rare insertions of a ∼5.3 kb element containing an ORF matching 'transpos*' in protein BLAST, plus a few more common insertions of a ∼450 bp element without coding potential (**a–b**). A dot plot (**c**) and multiple alignment of all alleles from the graph (**d**) reveal the presence of LTRs, and identify the ∼450 bp element as a solo-LTR. An exhaustive search (not limited to sSVs) reveals that the length of the element (**e**) is highly conserved, and it is mostly present in low copy number (**f–g**). The element is un-annotated because it is not present in the TAIR10 reference genome—only a solo-LTR is found (**h** and **j**). The element contains two long putative ORFs (**i**) without matches in genomes of closely related species (*e.g., Brassica rapa*) and but possible matches in potato and sweet orange (**j**). Maps were generated using public domain data from the Natural Earth project via the R package rnaturalearth.

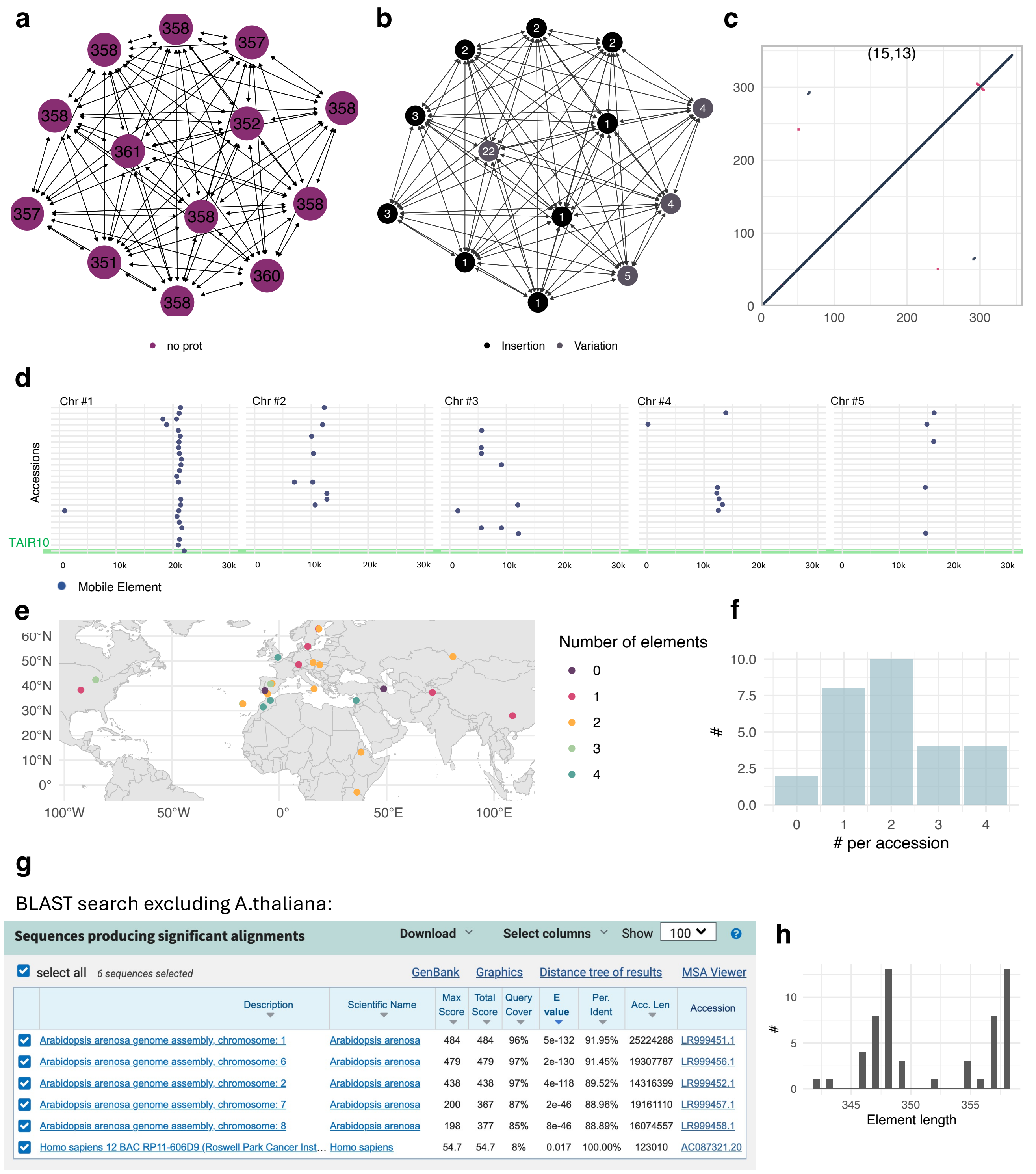

Description	Scientific Name	Max Score	Total Score	Query Cover	E value	Per. Ident	Acc. Len	Accession
Arabidopsis arenosa genome assembly, chromosome: 1	Arabidopsis arenosa	484	484	96%	5e-132	91.95%	25224288	LR999451.1
Arabidopsis arenosa genome assembly, chromosome: 6	Arabidopsis arenosa	479	479	97%	2e-130	91.45%	19307787	LR999456.1
Arabidopsis arenosa genome assembly, chromosome: 2	Arabidopsis arenosa	438	438	97%	4e-118	89.52%	14316399	LR999452.1
Arabidopsis arenosa genome assembly, chromosome: 7	Arabidopsis arenosa	200	367	87%	2e-46	88.96%	19161110	LR999457.1
Arabidopsis arenosa genome assembly, chromosome: 8	Arabidopsis arenosa	198	377	85%	8e-46	88.89%	16074557	LR999458.1
Homo sapiens 12 BAC RP11-606D9 (Roswell Park Cancer Inst…	Homo sapiens	54.7	54.7	8%	0.017	100.00%	123010	AC087321.20

Supplementary Fig. 17 | A putative novel mobile element family. A connected component from the graph of nestedness (Fig. 5) built on sSVs that have no overlap with annotated TEs. **a–b,** The component consists of many variable-frequency insertions of a short sequence of 351-361 bp without coding potential. The element is not repetitive (**c**) but is surrounded by putative target-site duplications. The element is low copy-number, but insertions are sometimes common (**d–f**); (**d**) contains all detected instances, not only those in sSVs). An NCBI BLAST search excluding *A. thaliana* (**g**) identified no matches except in the closely related *A. arenosa*. **h,** The length distribution of the element, again including all detected instances, not only those in sSVs. Maps were generated using public domain data from the Natural Earth project via the R package rnaturalearth.

5.3 Silencing of sSVs

We investigated silencing in sSVs using existing bisulfate sequencing data[53] for 12 of our accessions. For each accession, we remapped this raw data onto its corresponding genome and estimated the level of CG, CHG and CHH methylation for each SV in each accession (in case the variant was present) (see Methods). Focusing first on those *with* annotated TE content, we note the following (Supplementary Fig. 18):

- sSVs corresponding to complete TEs are most highly methylated, followed by those corresponding to TE fragments. sSVs containing TEs or TE fragments are more variable, consistent with a subset of these sSVs corresponding to un- or mis-annotated TEs (Supplementary Fig. 18a).
- For sSVs corresponding to complete TEs, methylation increases with frequency, indicating that older insertion are more highly methylated. For sSVs containing TEs or TE fragments, this pattern changes at high frequencies, which could reflect high-frequency presence alleles not being insertions of un- or mis-annotated elements, but rather deletions that happen to contain TEs(Supplementary Fig. 18b).
- Methylation is higher on sSVs that are part of the graph of nestedness (Fig. 5), consistent with methylation targeting the mobile-ome (Supplementary Fig. 18c).

Turning to sSVs *without* annotated TE content, we see that they behave similarly to sSVs containing TEs or TE fragments, but are on average less methylated, consistent with a smaller fraction of these sSVs corresponding to unannotated TEs (Supplementary Fig. 18b). Note in particular that sSVs that are part of the graph of nestedness are almost as highly methylated as previously annotated TEs, whereas those that are not tend to be completely unmethylated (Supplementary Fig. 18c).

Expression patterns from existing RNA-seq data remapped onto corresponding genomes for each accession[54] (Methods) are consistent with the methylation data: sequences in sSVs corresponding to complete TEs are barely expressed (except in pollen, where some TE expression is known to occur[55]), while the behavior of sequences in other sSVs is more variable (Supplementary Fig. 19). Especially sSVs without annotated TE content are highly variable, with a small subset, presumably corresponding to protein-coding genes, having evidence of high expression levels.

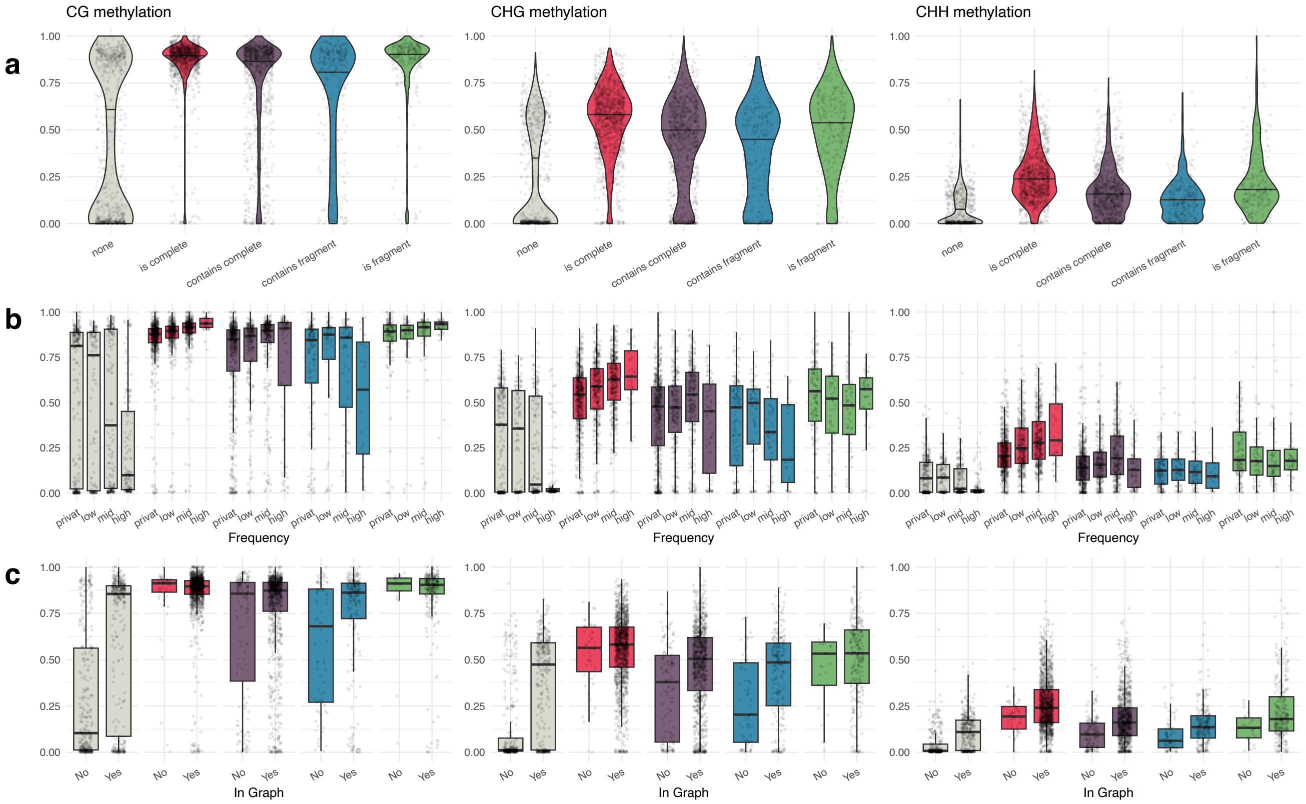

Supplementary Fig. 18 | Methylation of sSVs. **a,** Methylation levels of sSVs categorized by their TE content. Those corresponding to complete TEs or TE fragments are most highly methylated. **b,** Methylation levels of sSVs categorized by their TE content and frequency of presence ('private' = 1, 'low' = 2–3, 'mid' = 4–24, 'high' = 25–26). For those corresponding to complete TEs, methylation increases with frequency (which is correlated with age). **c,** Methylation levels of sSVs categorized by membership in the graph of nestedness (Fig. 5). Methylation levels are always higher for sSVs in the graph, and the difference for those without annotated TE content is striking. Methylation on SVs in 12 accessions was estimated using the BS-seq data from[53] mapped to their corresponding genomes (Methods). Only simple SVs are plotted. Each data point in the plots corresponds to the maximal methylation level of each sSV among the accessions where this sSV is present.

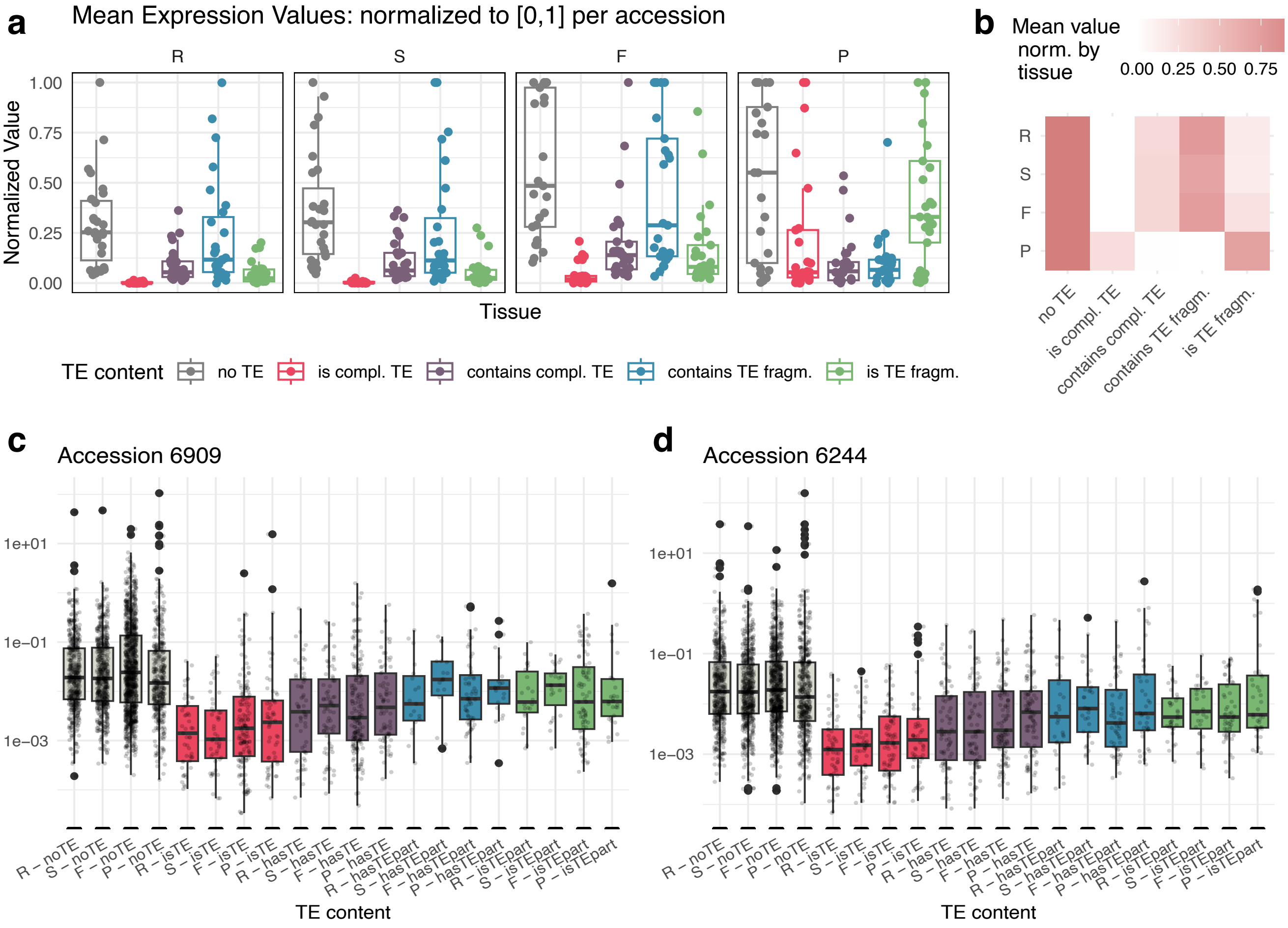

Supplementary Fig. 19 | Expression of sSV sequences. The distribution of expression levels as a function of different TE-coverage categories and tissues (R = rosette; S = seedling; F = flower; P = pollen), illustrating that sequences in sSVs that are more likely to be part of the mobile-ome have low expression. For each sSV, read counts were normalized by length. **a,** Each dot is the mean expression of sequences in sSVs in that category for an accession, using values normalized within accessions. **b,** Mean expression of sequences in sSVs for each category normalized within tissue. **c–d,** For two different accessions, the distribution of expression for each category and tissue is shown. Values have been normalized within tissue.

6 The gene-ome

6.1 Details about reconciling annotations and gene filtering

Our *de novo* annotation pipeline provided independent gene annotations for each accession (Methods). To define how gene annotations in different accessions correspond to each other, we made use of the pan-genome coordinate system (some studies approached this by finding matching orthologs[23], which is the only possibility for distantly related genomes, but can be suboptimal when working with similar genomes). Mapping annotations to the common pan-genome coordinates revealed that many genes had discordant annotations. Despite 83% of genetic loci having a one-to-one correspondence in the aligned genomes, gene model predictions varied considerably between accessions. While some of these differences likely represent genuine genetic variation, we lacked data to distinguish this from artifacts, and thus adopted a majority voting approach to harmonize annotations. The disagreements were of several types: (1) differences in exon-intron organization; (2) inconsistency in gene length; and (3) variability in the number of genes in a region. We resolved (3) by deciding to either merge or split genes based on a majority vote (Supplementary Fig. 20). We did not alter exon-intron organization and grouped genes into non-overlapping blocks for each strand to cover the maximum length (Supplementary Fig. 21). After identifying annotation groups on the pangenome coordinate, we compared the obtained gene and mRNA sequences between accessions. If, within a single annotation group, the sequences for accessions differed by more than 85% of their length or similarity, such genes were filtered out from the analysis. Such situations are associated with regions enriched with structural variations (SVs) or regions containing two or more haplotypes. Lastly, gene models that overlapped in the pan-genome coordinate system after the above-mentioned procedure were excluded from the analyses, ensuring that only one annotation group was considered. The resulting annotation groups were considered as genes and are referred to as 'genes' in this article.

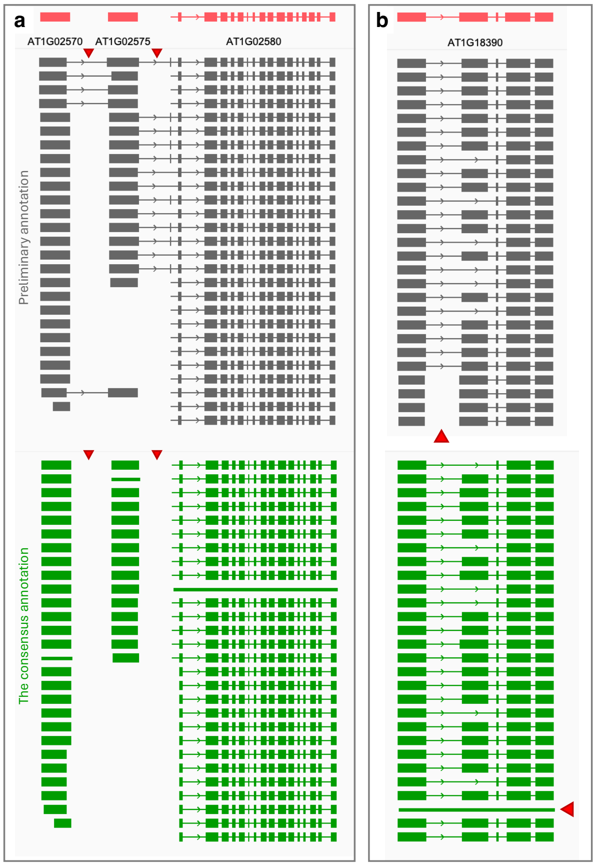

Supplementary Fig. 20 | Reconciling ambiguous gene annotation with majority voting. a, An example situation where the solution was to split gene annotations. Red triangles denote regions to solve. **b,** An example situation where the solution was to merge gene annotations. Red triangles denote the region to solve and the resultant merged gene.

After generating a consistent annotation in the pan-genome coordinates, we projected it back onto all accessions. If a

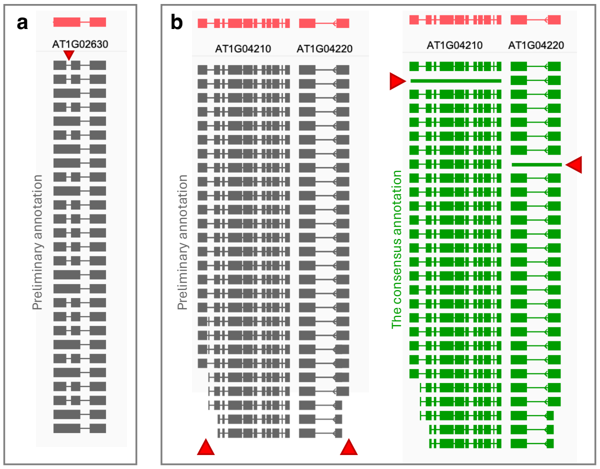

Supplementary Fig. 21 | Variation in gene models. A. Variation in exon-intron structure. Red triangles denote regions of exon-intron variation in the *de novo* annotations between accessions. **B.** Variation in gene length. Problems and how they were solved are indicated with red triangles. Red triangles on the gray plot indicate regions where transcript lengths vary between accessions. Red triangles on the green plot indicate regions of genes in the final annotation that cover the positions of all transcripts.

locus was present in an accession but not annotated by the *de novo* annotation, we still identified it as a gene, though without the exon-intron model. This procedure helped us to avoid underestimating the number of segregating genes (Supplementary Fig. 22). Supplementary Fig. 23 illustrates the final consensus annotation.

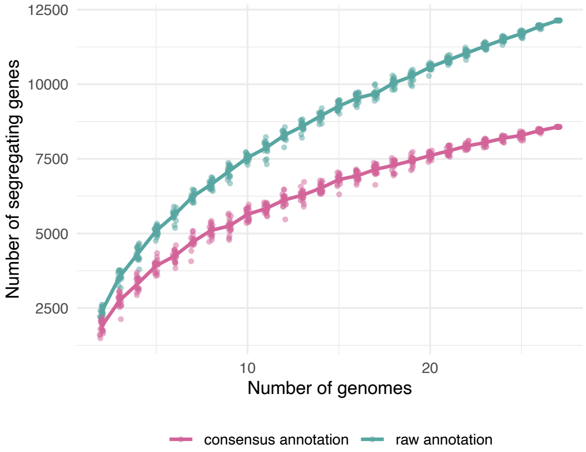

Supplementary Fig. 22 | Number of segregating genes. Comparison of the estimates based on the raw *de novo* annotation and on the final consensus gene annotation.

6.2 Genes and TEs

A. thaliana annotation groups were compared to TAIR10 annotations of protein-coding genes, pseudogenes, and TEs based on their position in the pan-genome coordinate system. Annotation groups without an overlap with TAIR10 annotations were marked as 'new genes'. We then separated genes into two main categories: protein-coding genes (TAIR10 and new) and TE genes (TAIR10 and new). To do so, we first classified the genes that had a TE-sequence content of >0.5 in at least one accession as TE genes, which resulted in assigning 4,885 genes as TE (Extended Data Fig. 5a). To make sure we did not miss any additional TE genes, we used UniProt[124] to look for amino acid similarity with genes encoding proteins known to participate in TE function. Annotation groups were classified as "TE genes" if their UniProt annotation met any

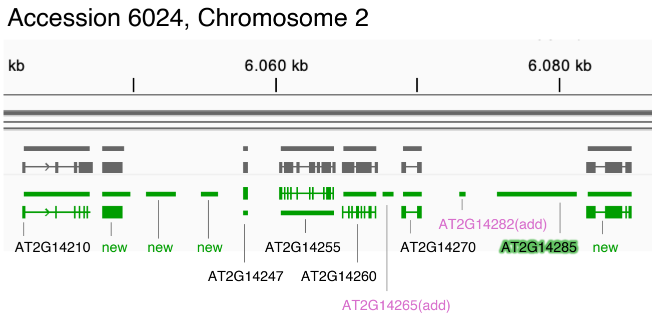

Supplementary Fig. 23 | An example of the final consensus annotation. This region of accession 6024 on chromosome 2 shows different sources of annotation: (i) TAIR10 genes, which were found in this accession (black names), (ii) TAIR10 genes, which were found in other accessions (black names with a green glow), (iii) TAIR10 genes, which were not found in any accession (purple names), (iv) new genes, which were found in this accession (denoted as 'new' with the gene model) and also in other accessions (denoted as 'new' without the gene model, a line only).

of the following conditions: UniProt protein names including transposon, retrotransposon, transposase, transposable, reverse transcriptase; GO terms including transposase, DNA transposition; their UniProt domain annotation including transposase, transposon, reverse transcriptase; had Pfam domains PF14223, PF03078, PF03732. This revealed 789 additional TE genes (Extended Data Fig. 5a) and gave a total of 5,674 TE genes - 17% of our annotated genes.

The rest of the genes (28,138) were classified as protein-coding. To further characterize protein-coding genes, their coding sequences were compared using DIAMOND's blastp module against UniProt DB (version 2024_06). The best hit in UniProt that shared at least 50% sequence identity over at least 50% of the *A. thaliana* gene length was further used. The functional and taxonomical annotation of UniProt hits were retrieved from uniprot KB using uniprot id-mapping service. Additional functional categories were defined based on protein names, keywords, domains, GO terms, and subcellular location annotations associated with the UniProt hit.

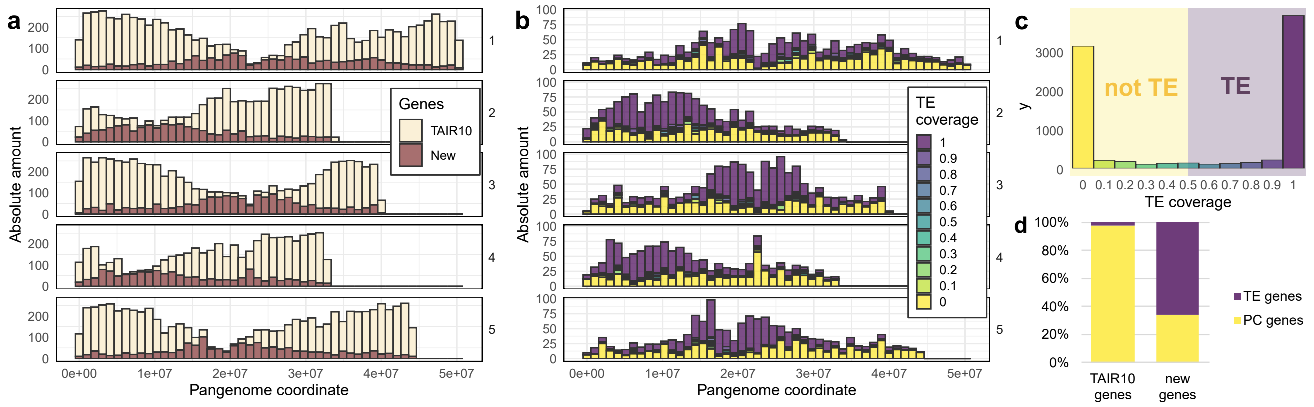

Supplementary Fig. 24 | TEs in *de novo* annotation. a, 'New genes' without correspondence to TAIR10 genes cluster around the centromere. **b,** The distribution of 'new genes' along the chromosome is explained by gene models with strong similarity to annotated TEs. **c,** The distribution of TE similarity among these gene models is bimodal, with 59% matching TEs. **d,** TE genes are strongly enriched among "new" genes.

6.3 New genes

We performed a more detailed analysis of 'new genes'—those that did not have a match in the TAIR10 gene annotation—to investigate their origin and characteristics.

First, we assessed the chromosomal distribution of new genes and found that they were enriched around the centromeres (Supplementary Fig. 24a), while TAIR10-annotated genes were depleted around centromeres. Grouping new genes based

on their TE-sequence content shows that the pericentromeric enrichment is largely due to genes with high TE content (Supplementary Fig. 24b,c). Thus, we find new protein-coding (non-TE) genes all across the chromosomes with slight depletion around centromeres. We could also see that new genes were, unsurprisingly, strongly enriched for TE genes (Supplementary Fig. 24d).

Next, we compared the presence-frequency of known (TAIR10) and new genes (Supplementary Fig. 25A) and found that, as expected, the great majority (94.2%) of TAIR10-annotated genes are fixed in the population, while the majority of new genes are not (82.9%). While new TE genes showed similar frequency distribution to new protein-coding genes, genes that were classified as TAIR10 TE genes are, surprisingly, often fixed (52.2%), which likely is due to the skewed representation of TAIR10 TE genes in our annotation: we only identified a small portion of annotated TE genes (565), and since our annotation pipeline relied on RNA expression, those are likely TE genes with higher-than-average expression. Since expression is markedly higher among fixed genes (Fig. 6i), this might explain an over-representation of fixed 'TAIR10 TE genes' genes in our annotation. Finally, high-frequency genes are more likely to have already been already annotated in TAIR10.

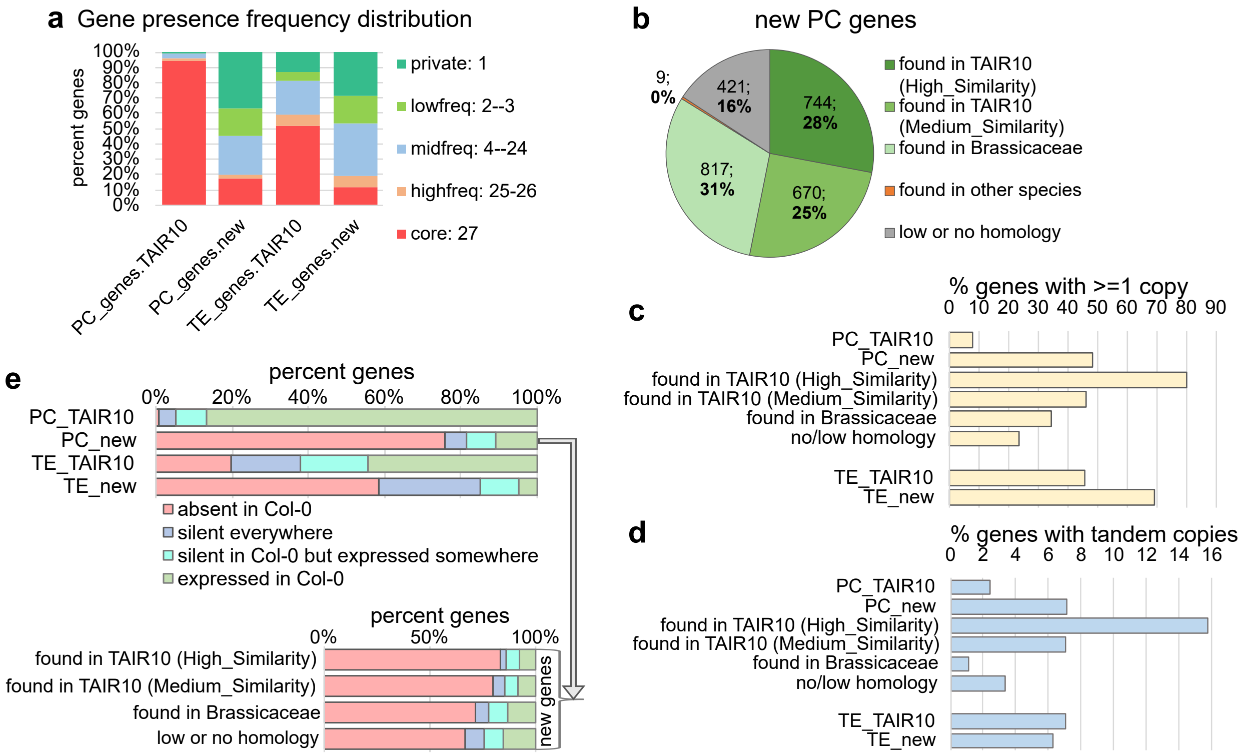

Supplementary Fig. 25 | 'New' genes. a, Presence-frequency for newly-annotated and TAIR10-annotated protein-coding and TE genes. **b,** New protein-coding genes grouped by amino acid sequence similarity to TAIR10 genes and UniProt genes best hit. High and medium similarity to TAIR10 gene corresponds to hits having either more than 90% or 45% sequence identity covering either more than 80 or 50% of the new PC, respectively. Hits to UniProt genes were considered if they share more than 45% sequence identity covering more than 50% of the new PC. For 'New PC' found only in UniProt, the groups were defined based on UniProt taxonomic annotation. **c,** Percent of genes in different categories that are duplicated in at least one accession. **d,** Percent of genes in different categories that are tandemly duplicated in at least one accession. Tandem duplications were defined as copies located within 10 kb from each other. Copy search was done using the simsearch tool in the Pannagram package. **e,** Investigating reasons for why a new gene may or may not be in the TAIR10 annotation: the locus is absent from Col-0 (red); the gene is silent in all accessions and tissues (blue); the gene is silent in Col-0, but expressed in at least one other accession (turquoise); the gene is expressed in Col-0 (green). An expression cutoff was used: locus-wide TPM > 0.25. Top: TAIR10- and newly annotated protein-coding and TE genes. Bottom: groups defined in **(b)**.

In order to identify the origin of the 'new' genes in our annotation, we searched for these genes in the genomes of TAIR10 (Col-0), other accessions, *A. lyrata*, other Brassicaceae species, and all other species using UniProt KB (Supplementary Fig. 25b). We categorized 'new' genes as 'TAIR10 high similarity' if they shared more than 80% sequence identity over 80% of the coding sequence length and as 'TAIR10 medium similarity' if they share between 45% and 80% sequence identity with the TAIR10 hit. A little more than a quarter, 28%, of previously unannotated protein-coding genes had a very high sequence

similarity (at least 80% identity) to TAIR10, indicating that they originated by recent gene duplication. Another quarter, 25%, had medium similarity (45-80% identity) suggesting more ancient gene duplications, or, alternatively, a recently duplicated gene modified by a large structural variation. Another 31% did not have a sequence match in TAIR10 but their sequences could be found in other accessions, or in other *Brassicaceae* species, including *A. lyrata*. Consistent with a likely origin via duplication, new genes were strongly enriched for being present in 2 or more copies (6.0-fold enrichment over TAIR10 genes, Supplementary Fig. 25c), with TAIR10-high-similarity genes showing the highest – 10.0-fold – enrichment. We can assume that a few of the duplications responsible for the creation of the new genes are tandem, as new genes showed a 3.0-fold enrichment in being in a tandem copy in at least one accession, compared to TAIR10-annotated genes, with high-similarity genes showing 6.5-fold enrichment (Supplementary Fig. 25d).

We next further investigated why the newly annotated genes were missing from the TAIR10 annotation. For most (75.9%) of the 'new' protein-coding genes, their genetic sequence is simply absent from the Col-0 genome (Supplementary Fig. 25e). New genes might also be missing from the TAIR10 annotation because they are not expressed in Col-0, but we were able to annotate them if they are expressed in other accessions, which was the case for 7.5% of new genes; About 45% of the new genes that are present also in Col-0 (the TAIR10 accession) are also expressed there—albeit at a low level. It is also worth noting that our annotation pipeline identified many new genes that are not expressed in any accession, *i.e.*, their identification had been solely due to sequence-based prediction (see Methods). From the categories of new genes, the TAIR10-high-similarity genes have the highest rate of the locus being absent from TAIR10 (Supplementary Fig. 25E, bottom), confirming our intuition that these are the most recently arising genes that stem from a structural change of inserting a new gene locus through gene duplication.

We have shown that many new genes are often physically absent from specific accessions, but even where present, the new genes are often not expressed and show signs of PRC2 and TE-like silencing (Supplementary Fig. 26a–d). New TE genes show very high levels of silencing, consistent with the idea that many old (fixed) (Supplementary Fig. 25a–d) TE genes manage to increase their frequency by becoming harmless and thus requiring less silencing than new and potentially more deleterious TE genes. This is also supported by our finding that new TE genes are strikingly more enriched in functional TE domains (Fig. 6g). Different categories of new protein-coding genes (shown in Supplementary Fig. 25b) show no significant differences in expression or silencing, with the exception for slightly lower H3K9me2 and CG methylation level on TAIR10-high-similarity genes (Supplementary Fig. 25e).

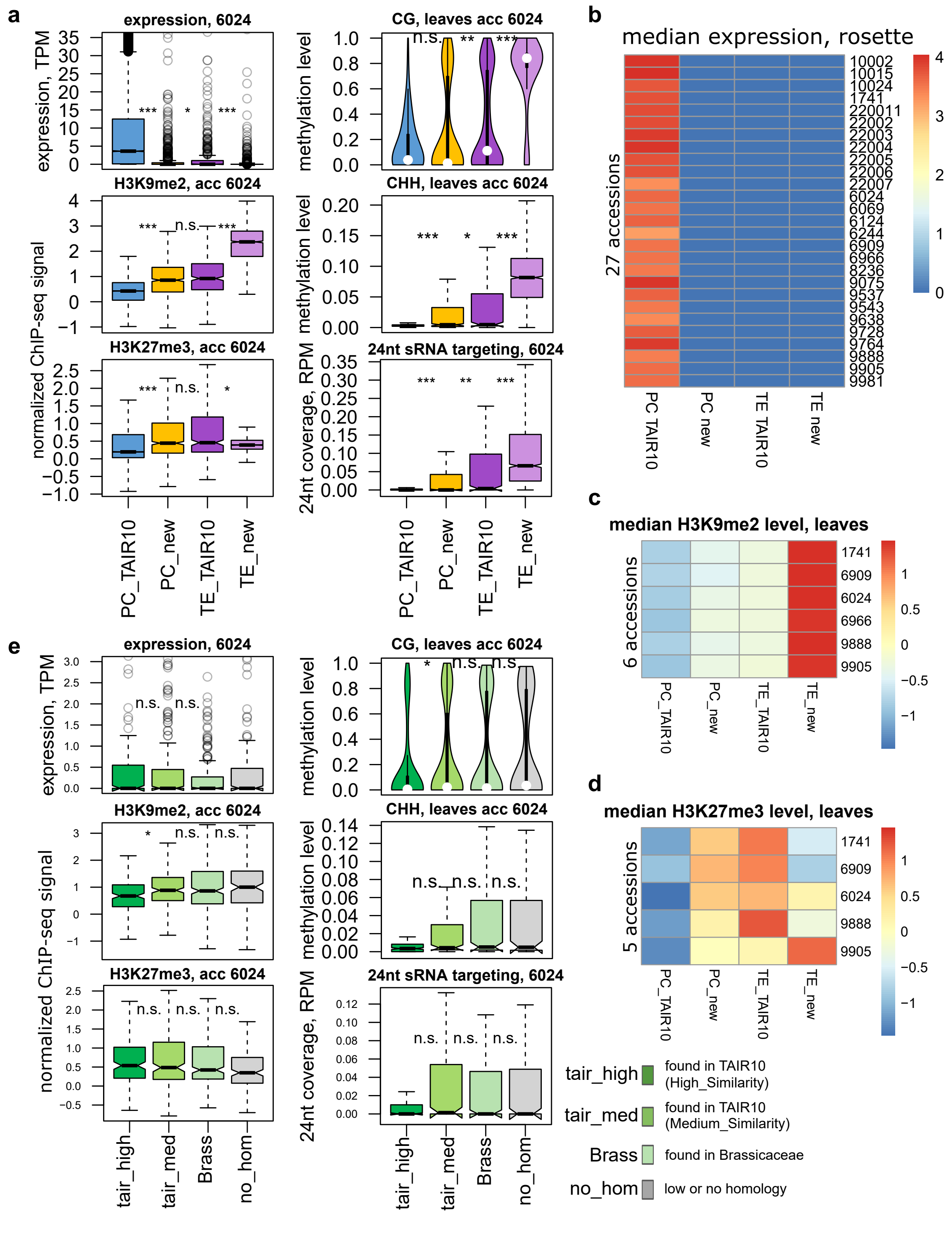

Supplementary Fig. 26 | Silencing of 'new" genes. **a,** For TAIR10 genes and new genes: Locus-wide RNA expression in 9-leaf rosette; levels of H3K9me2 and H3K27me3 in mature leaves; levels of CG and CHH methylation in mature leaves; and 24 nt sRNA levels in flowers[54]. Plots show accession 6024 only; other accessions showed similar patterns. **b,** Median expression of TAIR10 genes and new genes in 9-leaf rosette in 27 accessions. **c–d,** Median H3K9me2 and H3K27me3 levels in subsets of accessions for TAIR10 genes and new genes. **e,** For the 4 categories of new protein-coding genes defined in Supplementary Fig. 25B: locus-wide RNA expression in 9-leaf rosette; levels of H3K9me2 and H3K27me3 in mature leaves; levels of CG and CHH methylation in mature leaves; and 24 nt sRNA level in flowers[54]. Box and violin plots show accession 6024 only; other accessions showed similar patterns. Box plots show the median (center line), the 25th and 75th percentiles (box bounds), and the smallest and largest values within 1.5× the interquartile range (whiskers). Signals between gene categories were compared using two-sided Wilcoxon rank-sum test (***: $P < 10^{-10}$, **: $P < 10^{-5}$, *: $P < 10^{-2}$).

7 Errors and biases in SNP-calling

7.1 Results

To explore the sources of errors in SNP calling, we took advantage of the high-coverage, PCR-free short-read data that were used to correct PacBio reads during assembly. Briefly, for a pair of accessions, we first identified SNPs using a whole-genome alignment, then designated one of the genomes as a reference and called SNPs using short reads from the other genome. The process was repeated until each genome had been used as reference genome for every other genome in the sample. The results were compared using the whole-genome alignments as 'ground truth'.

Using standard parameters, we found that we could call far more SNPs than in our previous work (over 80% of those found in whole-genome alignment), but that the False Discovery Rate,

$$\mathtt{FDR} = \frac{\mathtt{FP}}{\mathtt{FP} + \mathtt{TP}},$$

was very high, ∼7%. A comprehensive investigation into the extent to which it is possible to decrease the number of false positives without increasing false negatives awaits investigation, but a closer look at the nature of these SNP-calling errors was already informative. As illustrated in Fig. 8A:

- 17.3% of SNPs identified in the whole-genome alignment were entirely missed by short read-based SNP-calling because they lie in regions not covered by reads because of mapping problems. This is the main reason SNP-calling underestimates polymorphism.
- 18.4% of SNPs called using short reads were pseudo-heterozygous (because the material analyzed was highly inbred and true heterozygous sites should not exist), almost entirely because the sample contained duplicated regions not found in the reference genome, leading to erroneous mapping of reads to regions that do not correspond to their true origin.
- 83.1% of false positive SNP calls were due to spurious read-mapping caused by various forms of polymorphism (not only SVs). A minor fraction (13.9%) were technical artifacts due to our two pipelines making different choices about local alignment.
- *Bona fide* false negative SNP-calls were either caused by read-mapping or local alignment problems, in roughly equal proportions.
- Other types of errors (including base-calling and random coverage) made trivial contributions.

It goes without saying that the precise numbers of SNP-calling errors will depend on the parameters used, but the qualitative conclusions will hold.

7.2 Methods and parameters

We used pairwise whole-genome alignments as a baseline ('ground truth') for comparison with short-read-based SNP calls. Mummer4[125] was employed for whole-genome alignment between corresponding chromosomes of accession pairs, and the `delta-filter` (`-1 -q -r`) was used to eliminate one-to-many and many-to-many redundant matches, followed by `show-snps` (`-THCr`) to extract variants. Only SNPs were retained for further examination.

For short-read-based SNP-calling, we used the PCR-free short reads generated for this study. Not only were these data generated from the same DNA preparations as was used for the PacBio CLR reads, but the coverage was also in general far higher than in previous work[34] (one accession, 22002, had less than 10x coverage, and was not used). Reads were mapped to each of the 27 genomes with BWA-MEM v0.7.17[116], followed by use of Picard tools to remove duplicates, and GATK HaplotypeCaller v4.3[126] to call variants with `gVCF` mode. Each of the 27 'reference' genomes was used to call SNPs in the remaining 25 samples. Variants were filtered by `QD < 2.0, FS > 60.0, MQ < 40.0, SOR > 4.0`, and genotypes with low quality or coverage were changed to missing with `GQ < 20 or DP < 3`. The VCFs were separated into pairwise comparisons for each combination of investigated accession and 'reference'. Since SNPs could be nested with other types variants (for example, with REF and ALT alleles of GTT and TTT,G, respectively), multi-allelic loci were first converted to bi-allelic with `bcftools norm -m -any` and the alternate alleles were realigned against the reference allele (transforming GTT/TTT to G/T) using vcfwave[127]. Heterozygous SNPs were extracted, and homozygous SNPs (MNPs here were considered as multiple SNPs) were retained for further evaluation.

The gVCF files were used to measure the genome fraction covered by short reads. Regions covered by fewer than 3 reads, or with GQ below 20, together with all heterozygous sites, were all considered not missing and excluded from the analysis

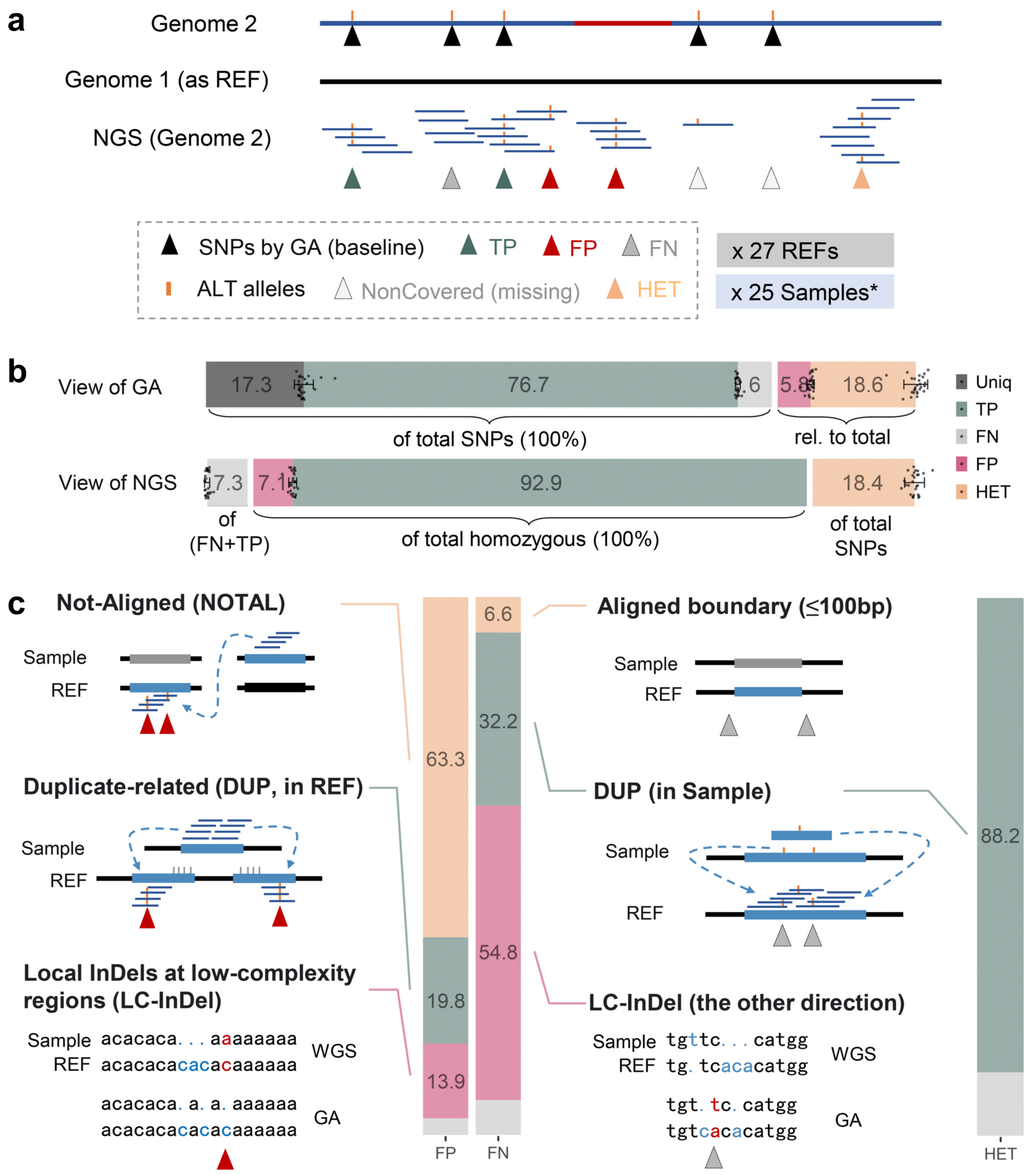

Supplementary Fig. 27 | Sources of errors in SNP-calling. a, Overview of the approach. Genome 1 is used as reference, and SNPs are identified both by whole-genome alignment to Genome 2 and by SNP-calling using short reads from Genome 2. The SNPs identified in the whole-genome alignment are treated as truth when compared with short read-based SNPs. The process was repeated using all 27 genomes as reference, and using 25 short read sets with sufficient coverage as samples. **b,** Sources of errors, in percent, for whole-genome alignment SNPs and short read-based SNPs (*cf.* Fig. 8a). Of the whole-genome alignment SNPs, 76.7% (on average) were also called using short reads, while 6% were FNs, and 17.3% were not called because the region was not covered by any read. Relative to the number of whole-genome alignment SNPs, 18.6% of short read-based SNP calls were heterozygous and 5.8% FPs. From the point-of-view of the short read-based SNPs, 7.1% of homozygous SNP calls were FPs. Heterozygous calls constituted 18.4% of total SNP calls, and the false negative rate, FNR $=$ FN$/($FN $+$ TP$)$ was 7.3%. **c,** Diagram of the sources of the three types of errors (FP, FN, and HET; see text for details, and Supplementary Figs. 28–31) for concrete examples).

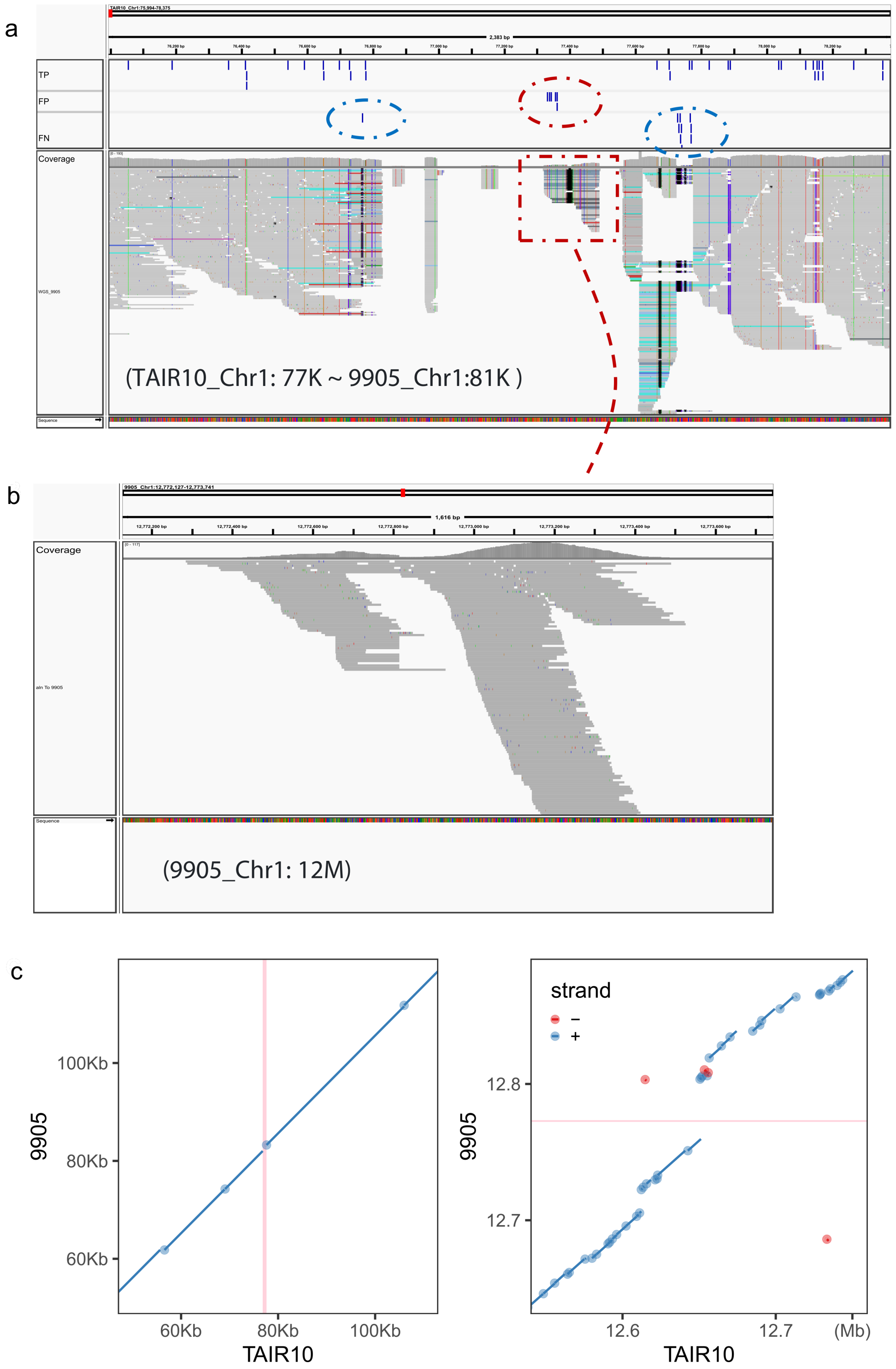

Supplementary Fig. 28 | Not Aligned (NOTAL) regions can cause both FP and FN calls. FP calls can be found in NOTAL regions, while FN calls can be found in flanking sequences. **a,** Screen shot from IGV showing FP (red circle) and FN (blue circle) SNPs, and the corresponding read-mapping, using WGA from 9905 to TAIR10 as an example. **b,** Aligning the mis-mapped reads (red square in **(a)**) to 9905 instead results in a well-mapped read to another non-collinear region. **c,**. Genome alignment showing that both the regions of **(a)** and **(b)** corresponded to NOTAL, while the vertical red lines represent the mis-mapped (non-collinear, gap) region in TAIR10, from which the reads can be well mapped to the region in 9905 (horizontal line). The diagonal lines are aligned regions, and the gaps are NOTAL regions.

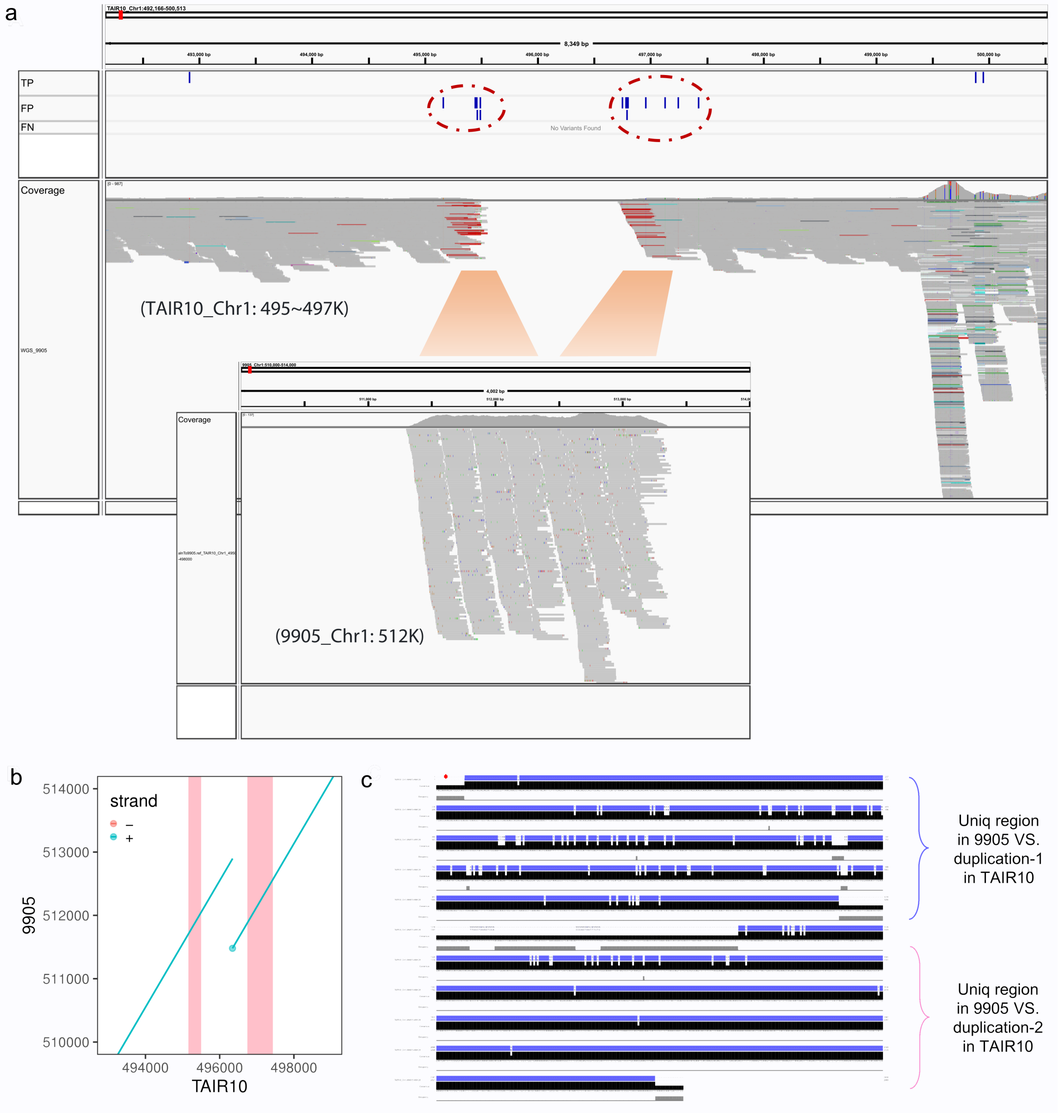

Supplementary Fig. 29 | How duplications introduce FP calls. FP calls can occur when there were more duplications in reference than in the new genome. **a,** Screen shot from Integrated Genome Viewer (IGV) shows the FP (framed by red circle) SNPs and corresponding reads mapped situations. The bottom IGV screen shot shows how these reads were mapped to the corresponding genome (9905), suggesting that the reads from one region were mapped separately to two different regions with a large gap between them. **b,** Further investigation showed that the region has only one copy in 9905 and two tandem duplications in TAIR10, resulting in a duplication event called in WGA, but not SNPs. The red regions indicate the two FP clusters based on TAIR10. **c,** This shows why the reads from one region of the 9905 genome were mapped separately to the early part of the first copy and the late part of the second copy of TAIR10. This is because these two regions were more identical to the one copy of 9905, so the reads could still be well aligned elsewhere in the TAIR10 genome, but not multi-mapped, resulting in false SNP calls.

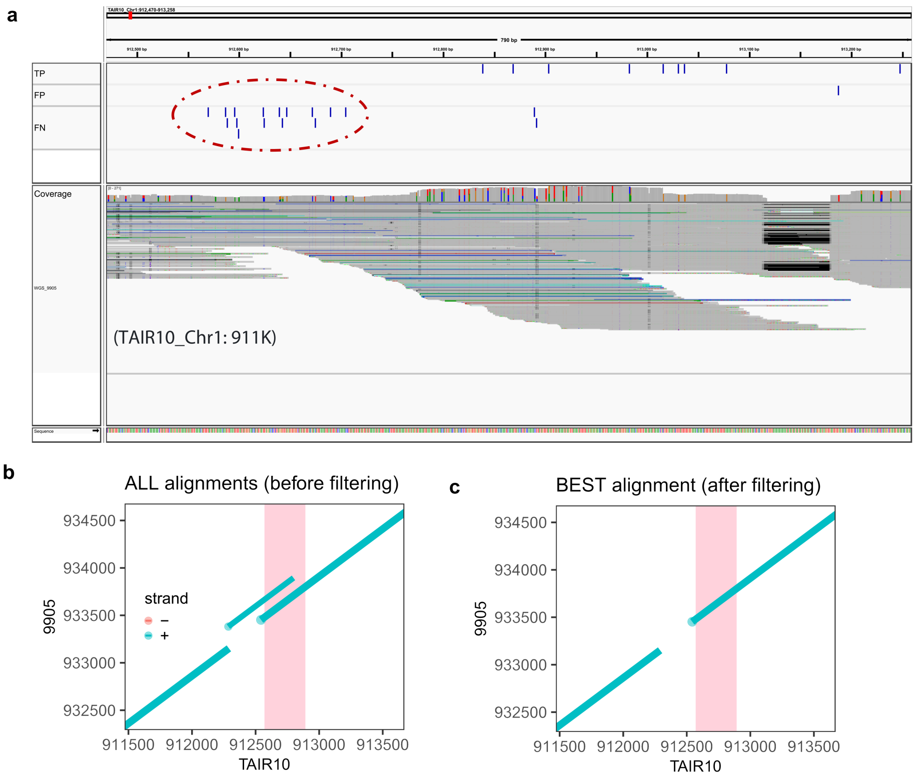

Supplementary Fig. 30 | How duplications introduce FN calls. More duplications in the new genome than in the reference can lead to FN SNPs. **a,** A screenshot from Integrated Genome Viewer (IGV) shows the FN SNPs (framed by the red circle) that can only be called in WGA and not in short-read mapping. **b,** Genome alignment showed that this region was duplicated in another short fragment in its own genome. The red region indicates the FN cluster based on mapping to TAIR10. **c,** However, in a WGA, it is easy to remove the short non-collinear alignment and use only the collinear alignment to call SNPs, whereas in NGS, this is impossible due to multi-mapping, and real differences are missed.

(Fig. 8a). Short read-based FPs were SNPs called with short reads but not from the whole-genome alignment or SNPs found with both approaches but with different ALT alleles. short read-based FNs were SNPs only called with a whole-genome alignment at sites covered by short reads.

To investigate the sources of SNP errors (Supplementary Fig. 27), the following steps were taken:

1. The filtered (`-1 -q -r`) ‘delta’ file produced by Nucmer was used to determine the ALN (Aligned, marks a block of aligned sequence between two genomes) and NOTAL (Not Aligned, highlights sections of a genome that did not align with the other) regions, and FP SNPs located within NOTAL regions were considered as ‘FP NOTAL’, and FN SNPs located within ALN regions but within 100 bp of NOTAL boundaries were considered ‘FN: Aligned boundary’.

2. The raw delta file including one-to-many and many-to-many alignments was processed using the command `show-snps` of Mummer4, and SNPs covered with multiple alignments (`[R]>0`) were retained to overlap with the FP calls to obtain the ‘FP: DUP’ category. To estimate the fraction of FNs caused by this category, whole-genome alignments (including inter-chromosomes) were produced with Nucmer, followed by `show-coords -THrcl` to obtain aligned corresponding regions.

3. The indels called from the WGA were used to investigate how much of the remaining NGS FP SNPs could be explained, while the indels called from the PCR-free short reads were used to assess that of FN SNPs. Erroneous (both FP and FN) SNPs within 5 bp of indels identified by the alternate technology were considered to be caused by local indels. These predominantly occurred in low complexity regions.

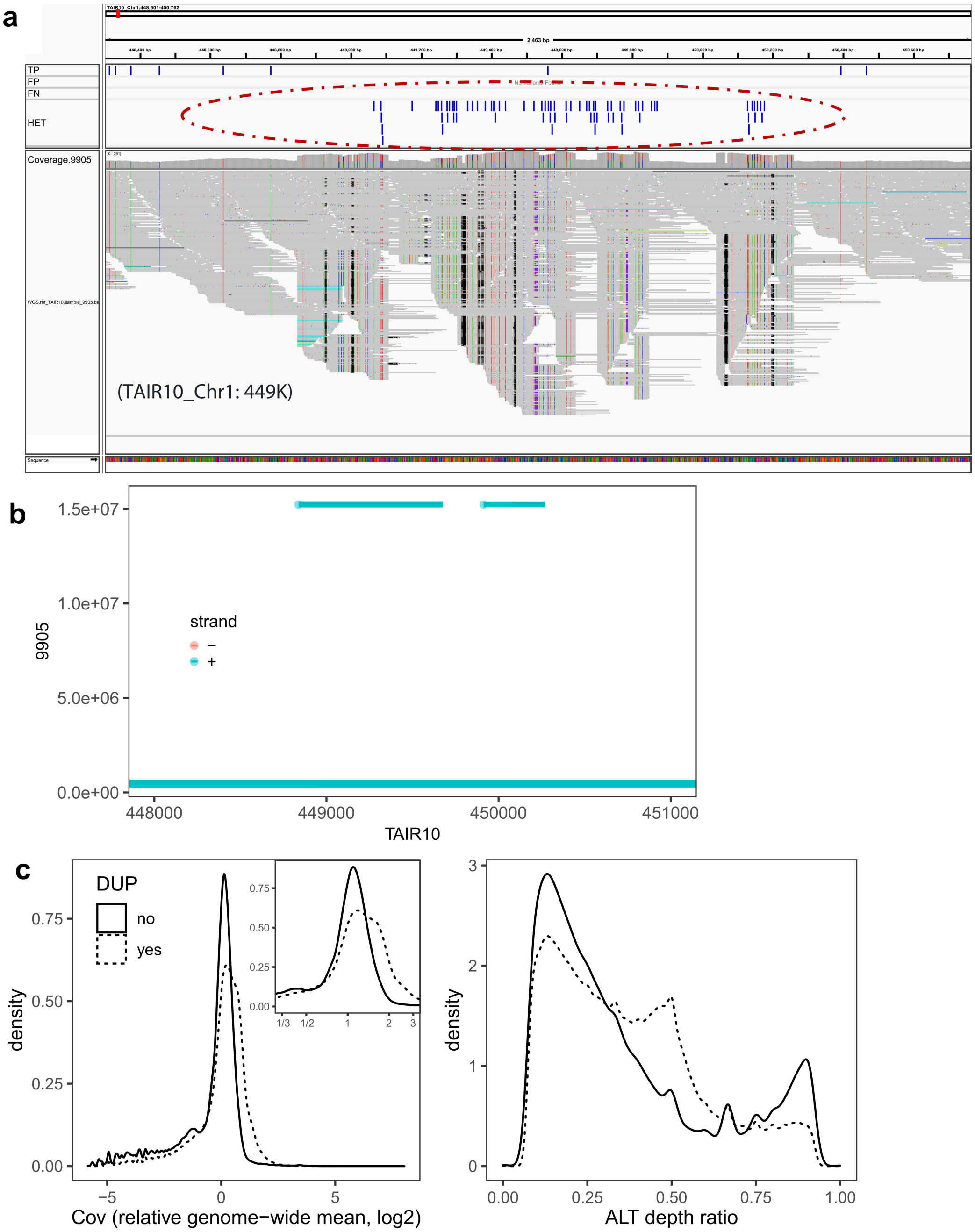

Supplementary Fig. 31 | Duplications contribute to the majority of mis-called heterozygous SNPs. Heterozygous (HET) SNPs can be called when there are more duplications in the new genome than in the reference. **a,** A screenshot from Integrated Genome Viewer (IGV) shows that heterozygous SNPs (framed by the red circle) were called in WGS due to more duplications in the sample genome than in the reference. The reads from the duplications were mapped to one location on TAIR10, leading to the identification of HET SNPs. **b,** Genome alignment showed that this large region is a segmental duplication in the new genome. **c,** The HET alleles from duplications that could be identified (DUP=yes, 88.2% of all HET variants) had higher read coverage, with the ALT allele depth ratio (reads with ALT alleles/total reads covered) enriched at 50% than the rest (that the 11.8% of HET variants that could not identify evidence of duplications).

8 Errors and biases in DNA methylation profiling

Many cytosines can be potentially missed, and some of their cytosine contexts (CG/CHG/CHH) are incorrect because of genetic differences between the TAIR10 reference and a focal genome (Supplementary Fig. 32a).

Even though for those that could be well aligned and with the same cytosine context, a DMR between accessions identified based on TAIR10 can be a FP (no significant differences based on corresponding genomes). To investigate this effect, BS-seq reads of each accession were mapped to TAIR10 (REF) and to the accession-specific genomes (OWN), and the unique and de-duplicated alignments were used to summarize the context-dependent methylation. Differentially methylated regions (DMRs) between two accessions were identified based on mapping to REF and mapping to OWN, respectively. For each comparison, say Acc1 vs Acc2, four analyses were performed, including Acc1_REF, Acc2_REF, Acc1_OWN and Acc2_OWN. Mappings from accession-specific analyses (*i.e.* Acc1_OWN and Acc2_OWN here) were aligned to the TAIR10 genome, and only aligned regions with a 1-to-1 correspondence were considered further. Only regions covered by at least 3 reads in both REF (either Acc1_REF or Acc2_REF) and OWN (either Acc1_OWN or Acc2_OWN) were retained, and DMRs between two accessions were identified based on REF (Acc1_REF vs Acc2_REF, DMR_REF) and OWN genomes (Acc1_OWN and Acc2_OWN, DMR_OWN). Cytosines were grouped into 100 bp non-overlapping windows, and a fast Fisher's exact test (`https://github.com/al2na/methylKit/issues/96`) was used to identify DMRs ($P \leq 0.01$) by summing all reads supporting methylation of all cytosines (allC) and total coverage in a given window. The analyses were repeated for individual cytosine contexts (CG, CHG, and CHH) (Supplementary Fig. 32b). Windows with zero read coverage (Cov $= 0$) in one accession, but over three methylated reads (mC ≥ 3) in the other, were manually assigned as DMRs ($P = 1E{-}4$). The same analysis was applied to both REF and OWN analysis, and for various contexts: all Cs (allC), only CG, CHG, and CHH.

To determine the enrichment overlapping with annotations of potential False Discovery DMRs (FDR_DMR) from REF-based analysis, continuous FDR_DMRs were first grouped together, and the obtained intervals in REF were used to measure the overlap with annotations. A permutation overlap analysis (same interval size and number in each chromosome were simulated for each genome comparison, with 100 repeats; implemented with `bedtools shuffle` was considered as background. The median fold change (in log2, 0 overlap was assigned to 0.2 for log calculation) of the observed annotation overlapping the 100 permutations was obtained as an indication of enrichment of each annotation in every genome comparison, and the enrichment of each annotation in all 10 sample comparisons (vs. the other one) were measured as a general trend (Supplementary Fig. 32c).

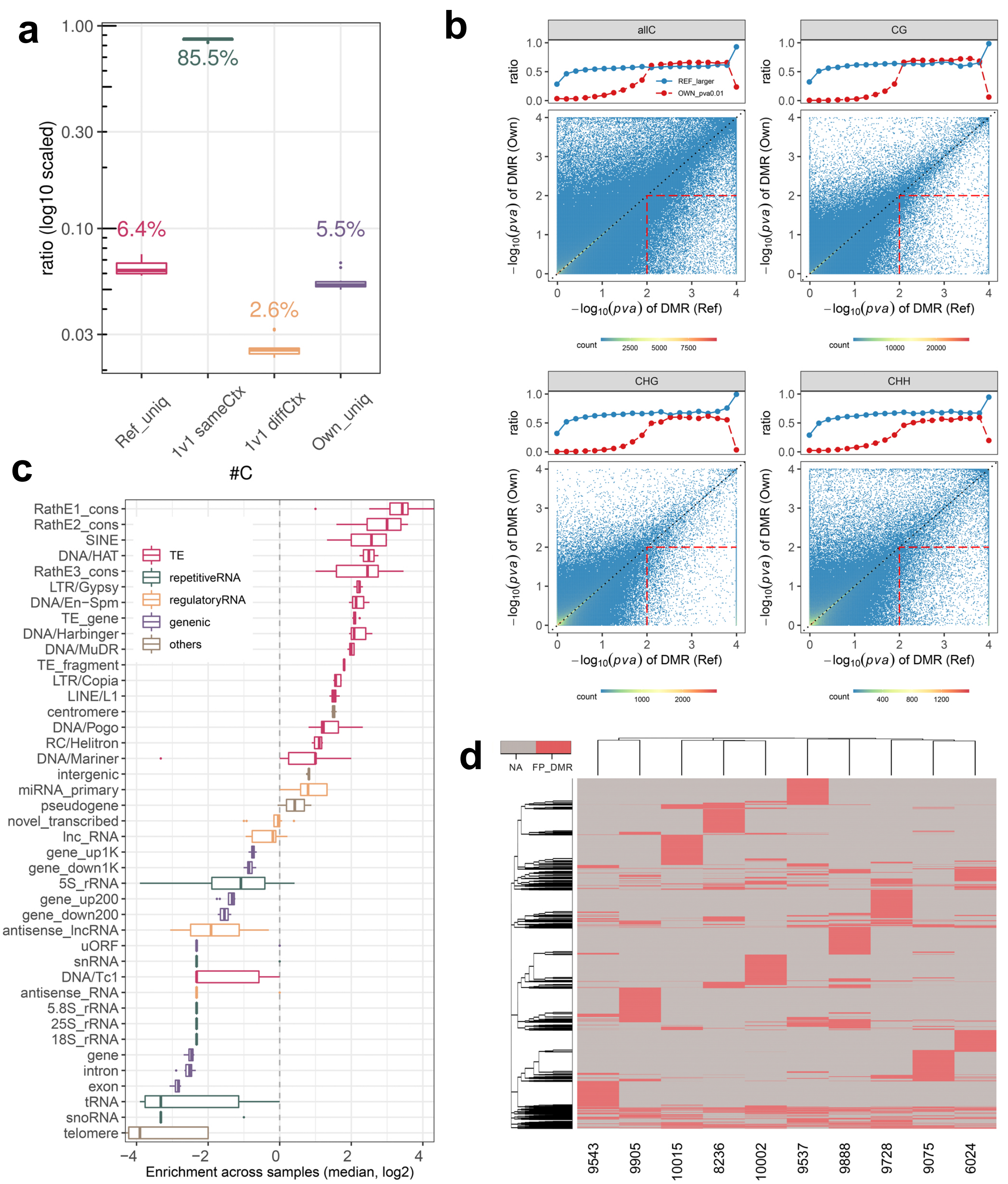

Supplementary Fig. 32 | Reference bias: methylation. a, Differences in cytosine content between TAIR10 and genomes newly assembled in this study due to sequence variation: '_uniq' indicates unaligned sequences from insertions, deletions, duplications, etc.; '1v1 sameCtx' represents aligned cytosines with conserved CG/CHG/CHH context; and '1v1 diffCtx' have aligned cytosines in a different context. Values are median across 11 samples. **b,** Differentially methylated regions (DMRs) between accessions were identified in 100-bp windows by mapping BS-seq reads to TAIR10 (x-axis) or to an accessions's own genome (y-axis). The red squares are FPs identified only when using the TARIR10 reference genome. Plots show results for comparison of all accessions to accession 6966. The blue line above shows the fraction of DMRs at a given p-value threshold (along the x-axis) with higher p-values in the TAIR10 reference analysis. The red line shows the fraction of reference-based DMRs that are also significant at $P < 0.01$ in the own-genome analysis. **c,** FP DMRs ($P < 0.01$ only in the reference-based analysis) are strongly enriched for TEs based on the annotations from the TAIR10. **d,** Most of the identified FP DMRs are unique to a specific accession.

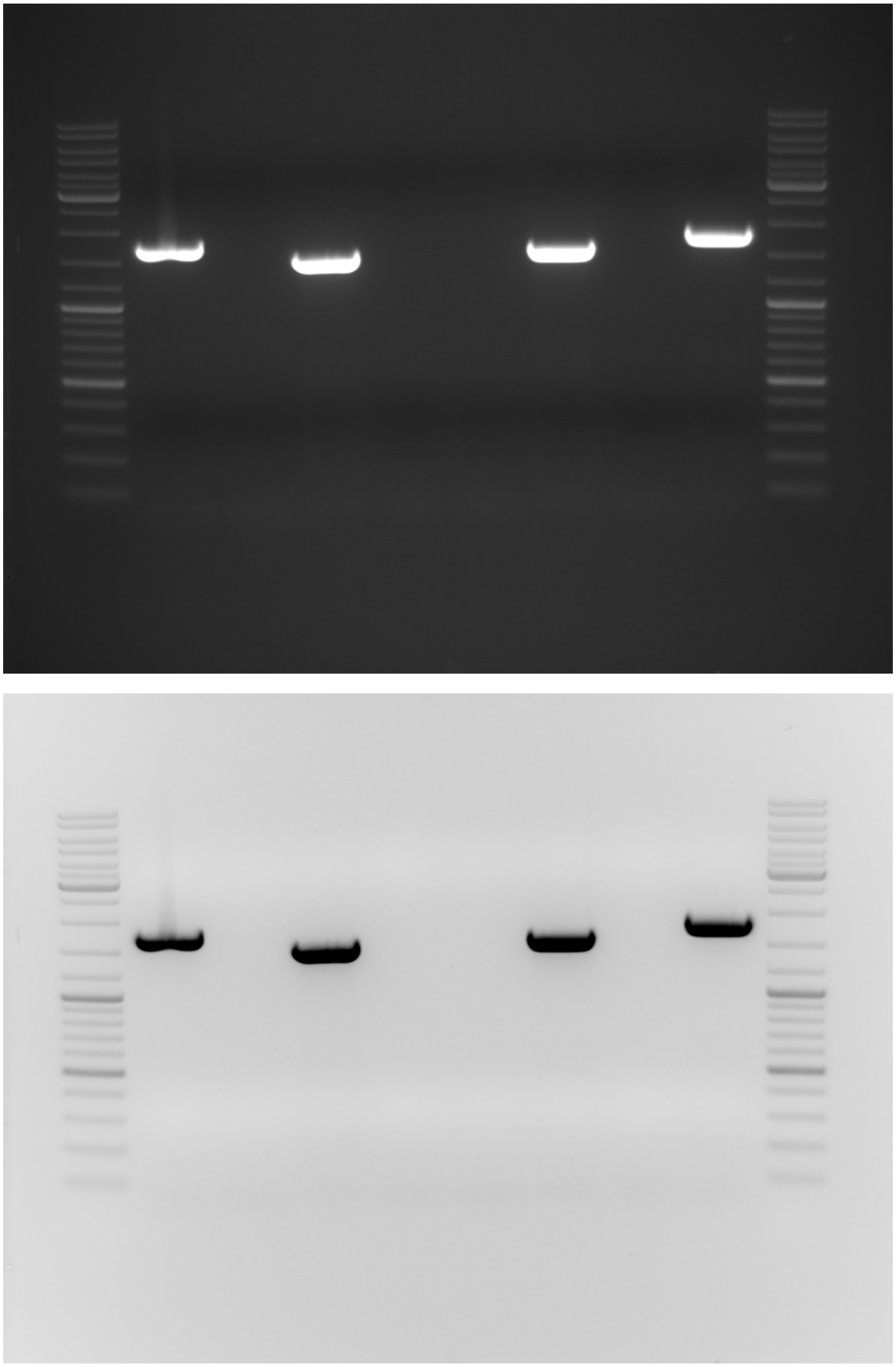

Supplementary Fig. 33 | Original (top) and inverted gel images corresponding to Supplementary Fig. 5

Supplementary-only References

114. Rhie, A. *et al.* Towards complete and error-free genome assemblies of all vertebrate species. *Nature* **592**, 737–746 (2021).

115. Lamesch, P. *et al.* The Arabidopsis Information Resource (TAIR): improved gene annotation and new tools. *Nucleic Acids Res.* **40**, D1202–10 (2012).

116. Li, H. Aligning sequence reads, clone sequences and assembly contigs with BWA-MEM. *arXiv* (2013). 1303.3997.

117. Li, H. *et al.* The sequence Alignment/Map format and SAMtools. *Bioinformatics* **25**, 2078–2079 (2009).

118. Marçais, G. & Kingsford, C. A fast, lock-free approach for efficient parallel counting of occurrences of k-mers. *Bioinformatics* **27**, 764–770 (2011).

119. Sun, H., Ding, J., Piednoël, M. & Schneeberger, K. findGSE: estimating genome size variation within human and arabidopsis using k-mer frequencies. *Bioinformatics* (2017).

120. Altschul, S. F., Gish, W., Miller, W., Myers, E. W. & Lipman, D. J. Basic local alignment search tool. *J. Mol. Biol.* **215**, 403–410 (1990).

121. Li, H. Minimap2: pairwise alignment for nucleotide sequences. *Bioinformatics* **34**, 3094–3100 (2018).

122. Stupar, R. M. *et al.* Complex mtDNA constitutes an approximate 620-kb insertion on *Arabidopsis thaliana* chromosome 2: implication of potential sequencing errors caused by large-unit repeats. *Proc. Natl. Acad. Sci. U. S. A.* **98**, 5099–5103 (2001).

123. Fields, P. D. *et al.* Complete sequence of a 641-kb insertion of mitochondrial DNA in the *Arabidopsis thaliana* nuclear genome. *Genome Biol. Evol.* **14**, evac059 (2022).

124. UniProt Consortium. UniProt: The universal protein knowledgebase in 2025. *Nucleic Acids Res.* **53**, D609–D617 (2025).

125. Marçais, G. *et al.* MUMmer4: A fast and versatile genome alignment system. *PLoS Comput. Biol.* **14**, e1005944 (2018).

126. Poplin, R. *et al.* Scaling accurate genetic variant discovery to tens of thousands of samples. *bioRxiv* 201178 (2018).

127. Song, B. *et al.* AnchorWave: Sensitive alignment of genomes with high sequence diversity, extensive structural polymorphism, and whole-genome duplication. *Proc. Natl. Acad. Sci. U. S. A.* **119** (2022).
