# Supplementary material for: A comparison of 27 Arabidopsis thaliana genomes and the path toward an unbiased characterization of genetic polymorphism
Source: Nat Genet. 2025 Aug 19;57(9):2289–301. doi: 10.1038/s41588-025-02293-0 (PMC12425826; doi:10.1038/s41588-025-02293-0)
Supplement: Supplementary file 2 — Reporting Summary [file 41588_2025_2293_MOESM2_ESM.pdf]

nature portfolio

Corresponding author(s): *Double-anonymous peer review submissions: write DAPR and your manuscript number here instead of author names.*

Last updated by author(s): *YYYY-MM-DD*

Reporting Summary

Nature Portfolio wishes to improve the reproducibility of the work that we publish. This form provides structure for consistency and transparency in reporting. For further information on Nature Portfolio policies, see our Editorial Policies and the Editorial Policy Checklist.

Statistics

For all statistical analyses, confirm that the following items are present in the figure legend, table legend, main text, or Methods section.

n/a	Confirmed	
☐	☒	The exact sample size (*n*) for each experimental group/condition, given as a discrete number and unit of measurement
☐	☒	A statement on whether measurements were taken from distinct samples or whether the same sample was measured repeatedly
☐	☒	The statistical test(s) used AND whether they are one- or two-sided<br>*Only common tests should be described solely by name; describe more complex techniques in the Methods section.*
☐	☒	A description of all covariates tested
☐	☒	A description of any assumptions or corrections, such as tests of normality and adjustment for multiple comparisons
☐	☒	A full description of the statistical parameters including central tendency (e.g. means) or other basic estimates (e.g. regression coefficient) AND variation (e.g. standard deviation) or associated estimates of uncertainty (e.g. confidence intervals)
☐	☒	For null hypothesis testing, the test statistic (e.g. *F*, *t*, *r*) with confidence intervals, effect sizes, degrees of freedom and *P* value noted<br>*Give P values as exact values whenever suitable.*
☒	☐	For Bayesian analysis, information on the choice of priors and Markov chain Monte Carlo settings
☒	☐	For hierarchical and complex designs, identification of the appropriate level for tests and full reporting of outcomes
☐	☒	Estimates of effect sizes (e.g. Cohen's *d*, Pearson's *r*), indicating how they were calculated

Our web collection on statistics for biologists contains articles on many of the points above.

Software and code

Policy information about availability of computer code

Data collection	No software was used.
Data analysis	Our code and scripts are available on GitHub:<br>1 https://github.com/Gregor-Mendel-Institute/1001Gplus_paper<br>2 https://github.com/iganna/pannagram<br>3 https://github.com/weigelworld/auto-asm<br>The following software was used (not all have versions; all are in paper):<br>Canu v1.71<br>Arrow v2.3.2<br>Pilon v1.22<br>Bionano Access v1.5<br>Bionano Solve v3.6<br>RagTag v1.1.1<br>pbmm2 v1.3.0<br>samtools v1.9<br>NucFreq v0.1<br>RepeatMasker v4.0.9<br>tRNAscan-SE v2.0.6<br>EDTA v1.9.7<br>Pannagram v2.0.0-beta<br>MAFFT (ref 94)

PGGB (Jan 2024)
VCF v1.54.0
NCBI BLAST server
Augustus v3.3.3
BUSCO v4.0.1
Liftoff v1.63
SNAP (ref 100)
Cufflinks (ref 101)
EvidenceModeler (ref 99)
blastp v 2.0.11
STAR v2.7.1
Subread v2.0.1
cutadapt v2.4
STAR (ref 104)
deeptools (ref 106)
bed-tools v.2.27.1
TrimGalore (https://github.com/FelixKrueger/TrimGalore)
Bismark (ref 109)
minimap2 v2.16
samtools v1.9
Jellyfish v2.3.0
findGSE (ref 116)
BLAST v2.2.29
UniProt DB (v. 2024_06).
Mummer4
BWA-MEM v0.7.17
Picard tools
GATK HaplotypeCaller v4.3
vcfwave
Nucmer
https://github.com/al2na/methylKit/issues/96

For manuscripts utilizing custom algorithms or software that are central to the research but not yet described in published literature, software must be made available to editors and reviewers. We strongly encourage code deposition in a community repository (e.g. GitHub). See the Nature Portfolio guidelines for submitting code & software for further information.

Data

Policy information about availability of data

All manuscripts must include a data availability statement. This statement should provide the following information, where applicable:

- Accession codes, unique identifiers, or web links for publicly available datasets
- A description of any restrictions on data availability
- For clinical datasets or third party data, please ensure that the statement adheres to our policy

Raw sequencing data (PacBio CLR and Illumina PCR-free short reads) and genome assemblies have been deposited in the European Nucleotide Archive (https://www.ebi.ac.uk/ena/browser/home) under project accession number PRJEB73474 (ERP158243). Illumina PCR-free short reads for 61 additional accessions used to investigate the contribution of satellite repeats can be accessed under project accession number PRJEB73476 (ERP158245).

BS-seq data from mature leaves of 14-leaf rosettes are from the 1001 Genomes Project (Kawakatsu et al, 2016) and are available under GEO accession number GSE43857.
ChIP-, RNA-, and sRNA-seq data were likewise previously published (Kornienko et al, 2023). ChIP-seq data from 14-leaf rosettes are available under GEO accession number GSE226682; RNA-seq data from seedlings, 9-leaf rosettes, flowers, and pollen are available under GEO accession number GSE226691; and sRNA-seq data from flowers are available under the GEO accession number GSE224571.

Several widely used public databases were used in the analyses:
NCBI Protein Blast (https://www.ncbi.nlm.nih.gov/protein), UniProtKB} (version 2024_06) (https://www.uniprot.org/), and
the TAIR10 genome annotation (\https://www.arabidopsis.org/).

Research involving human participants, their data, or biological material

Policy information about studies with human participants or human data. See also policy information about sex, gender (identity/presentation), and sexual orientation and race, ethnicity and racism.

Reporting on sex and gender	Work is on plants
Reporting on race, ethnicity, or other socially relevant groupings	Work is on plants
Population characteristics	Work is on plants

Recruitment	Work is on plants
Ethics oversight	Work is on plants

Note that full information on the approval of the study protocol must also be provided in the manuscript.

Field-specific reporting

Please select the one below that is the best fit for your research. If you are not sure, read the appropriate sections before making your selection.

☒ Life sciences ☐ Behavioural & social sciences ☐ Ecological, evolutionary & environmental sciences

For a reference copy of the document with all sections, see nature.com/documents/nr-reporting-summary-flat.pdf

Life sciences study design

All studies must disclose on these points even when the disclosure is negative.

Sample size	Sample was selected to cover global diversity; no conclusions depend on sample size.
Data exclusions	No data were excluded.
Replication	Please note that this study contains no experiments designed to test a specific hypothesis (like a drug trial). Replication was only used for a few types of measurements (e.g. the previously published ChIP-seq data) and the sample sizes used are given in the relevant figures.
Randomization	There are no experimental groups to randomize in this study.
Blinding	There are no experimental groups to be blind to in this study.

Reporting for specific materials, systems and methods

We require information from authors about some types of materials, experimental systems and methods used in many studies. Here, indicate whether each material, system or method listed is relevant to your study. If you are not sure if a list item applies to your research, read the appropriate section before selecting a response.

Materials & experimental systems

n/a	Involved in the study	
☒	☐	Antibodies
☒	☐	Eukaryotic cell lines
☒	☐	Palaeontology and archaeology
☒	☐	Animals and other organisms
☒	☐	Clinical data
☒	☐	Dual use research of concern
☐	☒	Plants

Methods

n/a	Involved in the study	
☒	☐	ChIP-seq
☒	☐	Flow cytometry
☒	☐	MRI-based neuroimaging

Dual use research of concern

Policy information about dual use research of concern

Hazards

Could the accidental, deliberate or reckless misuse of agents or technologies generated in the work, or the application of information presented in the manuscript, pose a threat to:

No	Yes	
☒	☐	Public health
☒	☐	National security
☒	☐	Crops and/or livestock
☒	☐	Ecosystems
☒	☐	Any other significant area

Experiments of concern

Does the work involve any of these experiments of concern:

No	Yes	
☒	☐	Demonstrate how to render a vaccine ineffective
☒	☐	Confer resistance to therapeutically useful antibiotics or antiviral agents
☒	☐	Enhance the virulence of a pathogen or render a nonpathogen virulent
☒	☐	Increase transmissibility of a pathogen
☒	☐	Alter the host range of a pathogen
☒	☐	Enable evasion of diagnostic/detection modalities
☒	☐	Enable the weaponization of a biological agent or toxin
☒	☐	Any other potentially harmful combination of experiments and agents

Plants

Seed stocks	No new seed stocks were collected: all are publicly avaiable.
Novel plant genotypes	None.
Authentication	DNA and RNA sequences were compared to public SNP data whenever possible.
